# Supplementary material for: New Janus Tricyclic Laddersiloxanes: Synthesis, Characterization, and Reactivity
Source: Molecules. 2023 Jul 27;28(15):5699. doi: 10.3390/molecules28155699 (PMC10420224; doi:10.3390/molecules28155699)
Supplement: Supplementary file 1 [file molecules-28-05699-s001.zip › molecules-2508063-supplementary-done.pdf]

molecules

Supplementary Materials

New Janus Tricyclic Laddersiloxanes: Synthesis, Characterization, and Reactivity

Yujia Liu [1,*], Midori Tokuda [1], Nobuhiro Takeda [1], Armelle Ouali [2,*] and Masafumi Unno [1,*]

[1] Department of Chemistry and Chemical Biology, Gunma University, 1-5-1 Tenjin-cho, Kiryu 376-8515, Japan; midori.tokuda@gmail.com (M.T.); ntakeda@gunma-u.ac.jp (N.T.)
[2] ICGM, Univ. Montpellier, CNRS, ENSCM, 1919 Route de Mende, 34293 Montpellier, CEDEX 05, France
* Correspondence: yliu@gunma-u.ac.jp (Y.L.); armelle.ouali@enscm.fr (A.O.); unno@gunma-u.ac.jp (M.U.); Tel.: +81-27-730-1230 (M.U.)

Table of contents **Pages**

1. Characterization data for synthetic compounds **6-14** S2

2. ^{1}H, ^{13}C, ^{29}Si, ^{1}H-^{1}H COSY and ^{1}H-^{13}C HSQC NMR spectra for synthetic compounds **6-15** S6

3. MALDI-TOF MS Spectra for synthetic compounds **6-15** S30

4. Thermogravimetry/Differential Thermal Analysis (TG/DTA) for compounds **6-14** S35

1. Characterization data for synthetic compounds 6-14

6-8-6 tricyclic laddersiloxane (6)

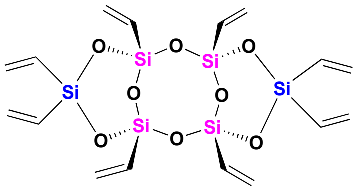

^1H NMR (600.17 MHz, $CDCl_3$): δ = 5.95-6.16 (m, 24H) ppm.
^{29}Si NMR (119.24 MHz, $CDCl_3$): δ = –35.21, –69.15 ppm.
^{13}C NMR (150.91 MHz, $CDCl_3$): δ = 128.65, 132.64, 133.07, 136.44, 137.39, 137.85 ppm.
MALDI-TOF MS (m/z): 514.96 ($[M+H]^+$, calcd 514.02).
Elemental analysis: Calcd for $C_{16}H_{24}O_8Si_6$: C, 37.47; H, 4.72. Found: C, 37.15; H, 4.79.

6-8-6 tricyclic laddersiloxane (7)

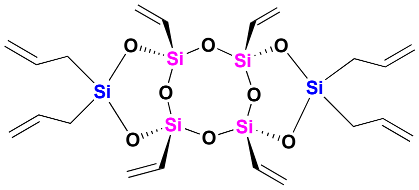

^1H NMR (600.17 MHz, $CDCl_3$): δ = 1.74 (d, *J* = 7.90 Hz, 4H), 1.80 (d, *J* = 7.90 Hz, 4H), 4.93-5.04 (m, 8H), 5.74-5.86 (m, 4H), 5.95 (dd, *J* = 20.62, 14.43 Hz, 4H), 6.07 (dd, *J* = 20.62, 4.12 Hz, 4H), 6.14 (dd, *J* = 14.43, 4.12 Hz, 4H) ppm.
^{29}Si NMR (119.24 MHz, $CDCl_3$): δ = –15.78, –69.17 ppm.
^{13}C NMR (150.91 MHz, $CDCl_3$): δ = 22.23, 23.25, 115.99, 116.06, 128.60, 131.45, 131.58, 137.83 ppm.
MALDI-TOF MS (m/z): 570.76 ($[M+H]^+$, calcd 570.08).
Elemental analysis: Calcd for $C_{20}H_{32}O_8Si_6·H_2O$: C, 40.92; H, 5.84. Found: C, 40.77; H, 5.85.

6-8-6 tricyclic laddersiloxane (8)

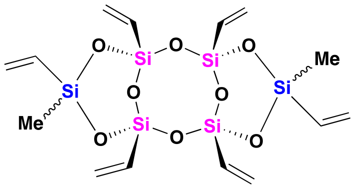

^1H NMR (600 .17 MHz, $CDCl_3$): δ = 0.31-0.38 (m, 6H), 5.94-6.13 (m, 18H) ppm.
^{29}Si NMR (119.24 MHz, $CDCl_3$): δ = –19.29, –19.39, –19.64, –19.77, –69.19, –69.25 ppm.
^{13}C NMR (150.91 MHz, $CDCl_3$): δ = –1.09, –0.82, –0.79, –0.73, 128.68, 128.76, 128.85, 128.89, 134.16, 134.40, 134.85, 134.93, 135.03, 135.10, 135.90, 136.16, 137.63, 137.66, 137.73, 137.79 ppm.
MALDI-TOF MS (m/z): 486.89 ($[M+H]^+$, calcd 486.02).
Elemental analysis: Calcd for $C_{14}H_{24}O_8Si_6·0.5H_2O$: C, 33.78, H, 5.06. Found: C, 33.75; H, 5.03.

6-8-6 tricyclic laddersiloxane (9)

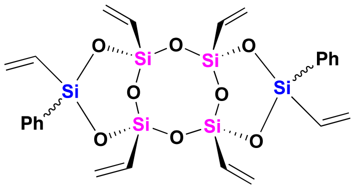

^{1}H NMR (600.17 MHz, $CDCl_3$): δ = 5.33 (dd, *J* = 19.59, 15.12 Hz, 0.33H), 5.39 (dd, *J* = 14.78, 4.47 Hz, 0.33H), 5.52 (dd, *J* = 19.59, 4.47 Hz, 0.33H), 5.89-6.24 (m, 16.68H), 6.29 (dd, *J* = 19.93, 14.78 Hz, 0.33H), 6.99 (t, *J* = 7.90 Hz, 1H), 7.22-7.24 (m, 0.5H), 7.35-7.47 (m, 6H), 7.56 (dd, *J* = 7.90, 1.37 Hz, 1H), 7.66 (dd, *J* = 7.90, 1.37 Hz, 0.5H), 7.76 (dd, *J* = 7.90, 1.37 Hz, 1H) ppm.
^{29}Si NMR (119.24 MHz, $CDCl_3$): δ = –33.86, –33.96, –34.02, –34.38, –68.40, –68.86, –69.00, –69.17 ppm.
^{13}C NMR (150.91 MHz, $CDCl_3$): δ = 127.84, 127.94, 128.04, 128.08, 128.52, 128.74, 130.57, 130.63, 130.67, 130.73, 131.62, 132.14, 132.57, 132.80, 133.48, 133.65, 133.89, 133.94, 133.99, 134.01, 134.22, 134.40, 136.37, 136.54, 137.16, 137.46, 137.96, 138.02 ppm.
MALDI-TOF MS (m/z): 634.78 ([M+Na]$^+$, calcd 635.03), 650.71 ([M+K]$^+$, calcd 651.00).

6-8-6 tricyclic laddersiloxane (10):

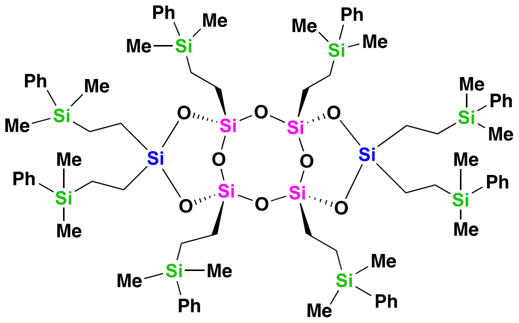

^{1}H NMR (600.17 MHz, $CDCl_3$): δ = 0.19-0.28 (m, 48H), 0.52-0.99 (m, 32H), 7.27-7.37 (m, 24H), 7.44-7.49 (m, 16H) ppm.
^{29}Si NMR (119.24 MHz, $CDCl_3$): δ = –1.12, –1.18, –1.31, –7.94, –55.06 ppm.
^{13}C NMR (150.91 MHz, $CDCl_3$): δ = –3.50, –3.47, –3.25, 5.08, 6.46, 6.60, 6.68, 6.92, 6.96, 127.69, 127.81, 127.91, 128.85, 129.03, 133.71, 133.84, 133.94, 134.04, 138.99, 139.07, 139.39 ppm.
MALDI-TOF MS (m/z): 1623.16 ([M+Na]$^+$, calcd 1623.56).
Elemental analysis: Calcd for $C_{80}H_{120}O_8Si_{14}\cdot0.5H_2O$: C, 59.28; H, 7.59. Found: C, 59.00; H, 7.61.

<u>**6-8-6 tricyclic laddersiloxane (11):**</u>

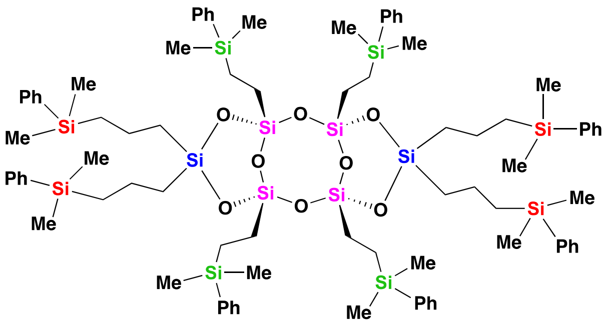

^{1}H NMR (600.17 MHz, CDCl$_3$): δ = 0.22 (s, 12H), 0.24 (s, 12H), 0.25 (s, 24H), 0.55-0.82 (m, 32H), 1.40- 1.43 (m, 8H), 7.31-7.36 (m, 24H), 7.47-7.49 (m, 16H) ppm.

^{29}Si NMR (119.24 MHz, CDCl$_3$): δ = –1.16, –3.80, –3.91, –8.46, –55.41 ppm.

^{13}C NMR (150.91 MHz, CDCl$_3$): δ = –3.48, –3.46, –2.85, –2.81, 5.04, 6.89,17.27, 17.38, 19.86, 20.02, 20.38, 20.52, 127.85, 127.87, 127.91, 128.88, 128.95, 129.02, 133.60, 133.65, 133.71, 138.98, 139.54, 139.60 ppm.

MALDI-TOF MS (m/z): 1680.35 ([M+Na]$^+$, calcd 1680.63)

Elemental analysis: Calcd for $C_{84}H_{128}O_8Si_{14}$: C, 60.81; H, 7.78. Found: C, 60.47; H, 7.91.

<u>**6-8-6 tricyclic laddersiloxane (12):**</u>

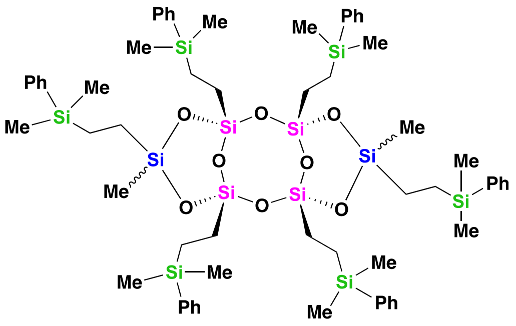

^{1}H NMR (600.17 MHz, CDCl$_3$): δ = 0.10-0.26 (m, 42H), 0.54-0.78 (m, 24H), 7.31-7.34 (m, 18H), 7.46-7.49 (m, 12H) ppm.

^{29}Si NMR (119.24 MHz, CDCl$_3$): δ = –1.10, –1.14, –6.19, –6.36, –6.82, –54.95, –54.99, –55.31, –55.39 ppm.

^{13}C NMR (150.91 MHz, CDCl$_3$): δ = –3.51, –3.48, –3.26, –1.64, –1.49, –1.08, 4.93, 4.99, 6.56, 6.68, 6.81, 6.95, 7.27, 8.83, 9.01, 9.41, 127.70, 127.84, 127.90, 128.87, 128.92, 128.96, 129.01, 133.70, 133.78, 133.86, 133.96, 138.96, 139.02, 139.08, 139.24, 139.44 ppm.

MALDI-TOF MS (m/z): 1327.16 ([M+Na]$^+$, calcd 1327.42), 1345.17 ([M+K]$^+$, calcd 1345.40).

Elemental analysis: Calcd for $C_{62}H_{96}O_8Si_{12}\cdot 2H_2O$: C, 55.47; H, 7.51. Found: C, 55.49; H, 7.51.

6-8-6 tricyclic laddersiloxane (13):

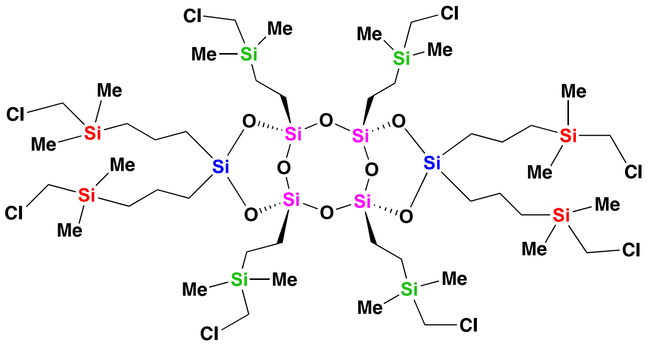

^{1}H NMR (600.17 MHz, CDCl$_3$): δ = 0.095-0.11 (m, 48H), 0.60-0.79 (m, 32H), 1.43-1.49 (m, 8H), 2.770 (s, 4H), 2.774 (s, 4H), 2.79 (s, 8H) ppm.

^{29}Si NMR (119.24 MHz, CDCl$_3$): δ = 5.37, 2.99, 2.93, –8.58, –55.58 ppm.

^{13}C NMR (150.91 MHz, CDCl$_3$): δ = –4.96, –4.39, –4.32, 4.76, 4.94, 17.06, 17.18, 17.96, 18.12, 20.39, 20.49, 29.94, 30.44 ppm.

MALDI-TOF MS (m/z): 1460.86 ([M+Na]$^+$, calcd 1460.18).

Elemental analysis: Calcd for $C_{44}H_{104}Cl_8O_8Si_{14}{\cdot}H_2O$: C, 36.29; H, 7.34. Found: C, 34.94; H, 7.30.

6-8-6 tricyclic laddersiloxane (14):

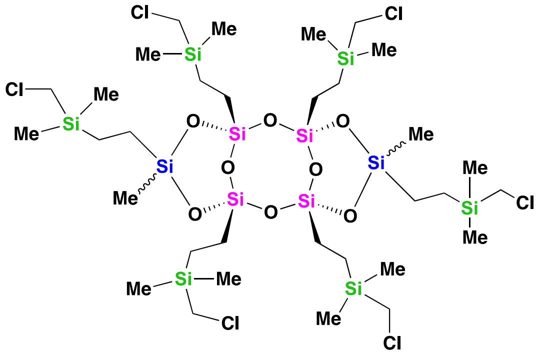

^{1}H NMR (600.17 MHz, CDCl$_3$): δ = 0.10-0.25 (m, 42H), 0.59-0.70 (m, 24H), 2.78-2.79 (m, 12H) ppm.

^{29}Si NMR (119.24 MHz, CDCl$_3$): δ = 5.61, 5.40, 5.20, –6.17, –6.57, –6.92, –55.12, –55.22, –55.58 ppm.

^{13}C NMR (150.91 MHz, CDCl$_3$): δ = –5.00, –4.80, –4.30, –1.67, –1.55, –1.29, –0.98, 4.58, 4.63, 4.69, 4.75, 4.83, 4.86, 4.92, 8.17, 8.69, 9.07, 9.25, 29.92, 30.02, 30.10 ppm

MALDI-TOF MS (m/z): 1162.79 ([M+Na]$^+$, calcd 1162.09).

Elemental analysis: Calcd for $C_{32}H_{78}Cl_6O_8Si_{12}{\cdot}2H_2O$: C, 32.66; H, 7.02. Found: C, 32.49; H, 6.98.

2. ^{1}H, ^{13}C, ^{29}Si, ^{1}H-^{1}H COSY and ^{1}H-^{13}C HSQC NMR Spectra for synthetic compounds 6-15

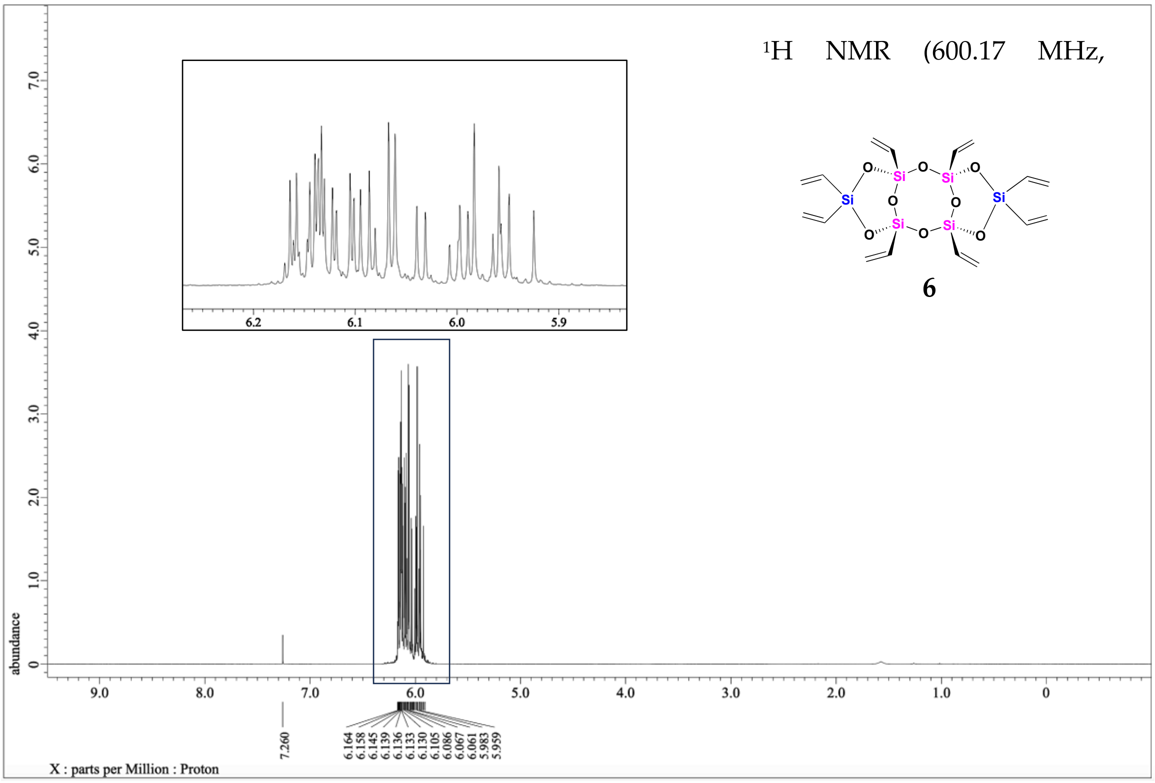

Figure S1. ^{1}H NMR spectrum for **6**.

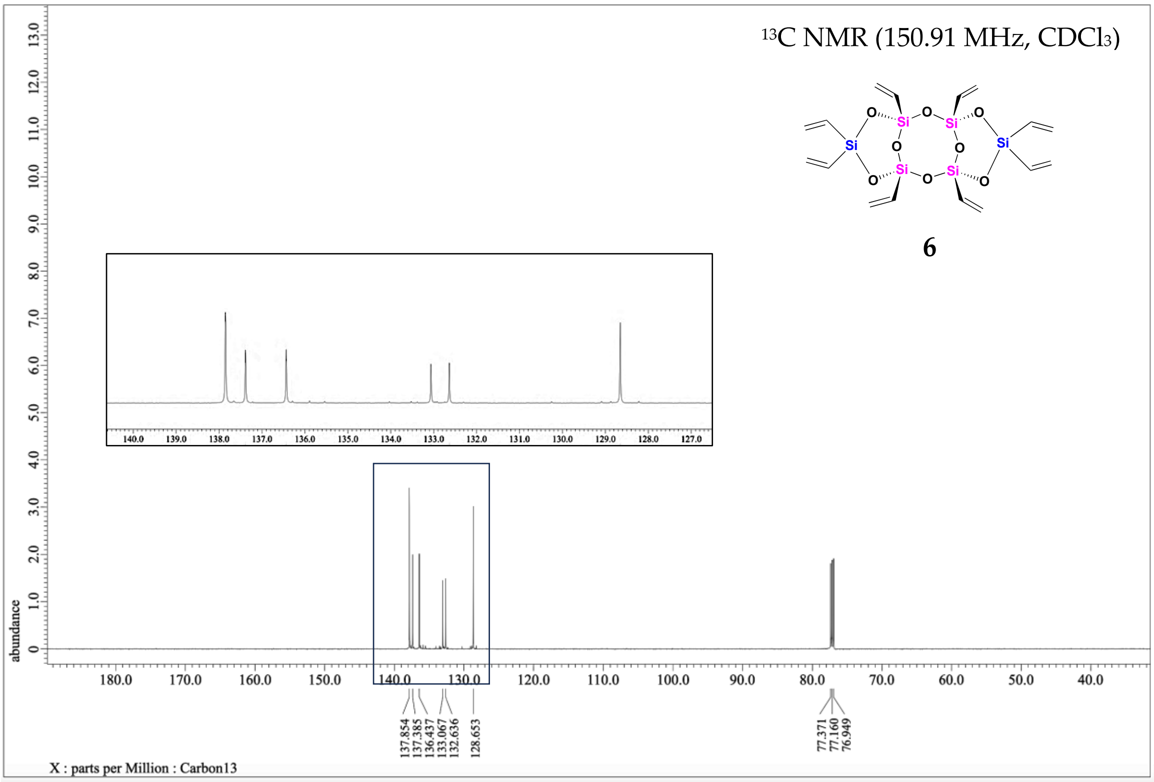

Figure S2. ^{13}C NMR spectrum for **6**.

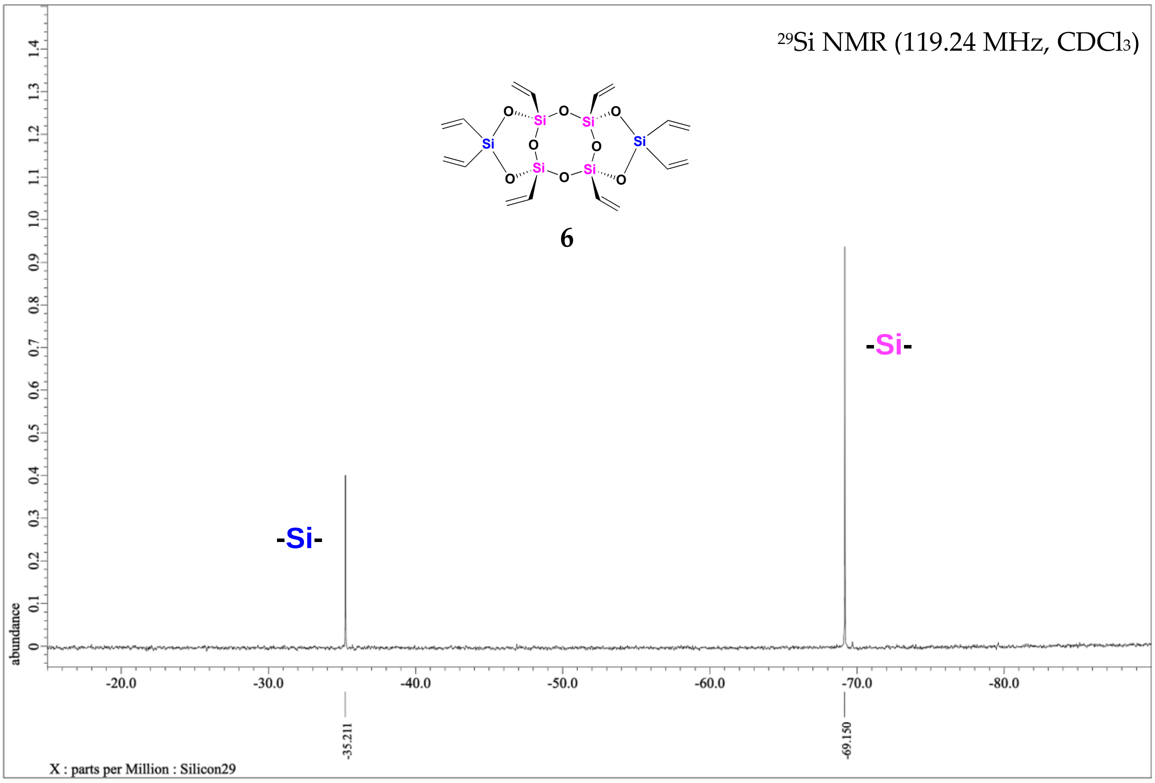

Figure S3. ^{29}Si NMR spectrum for **6**.

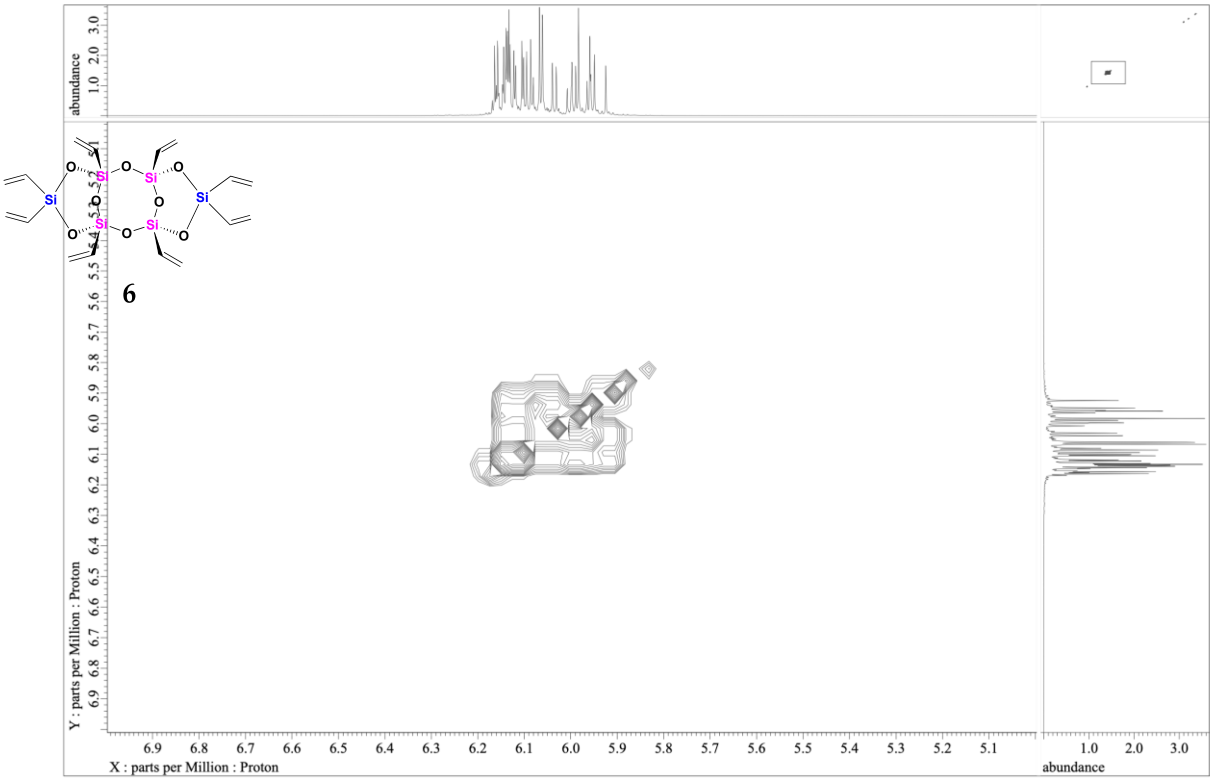

Figure S4. ^{1}H-^{1}H COSY NMR spectrum for **6**.

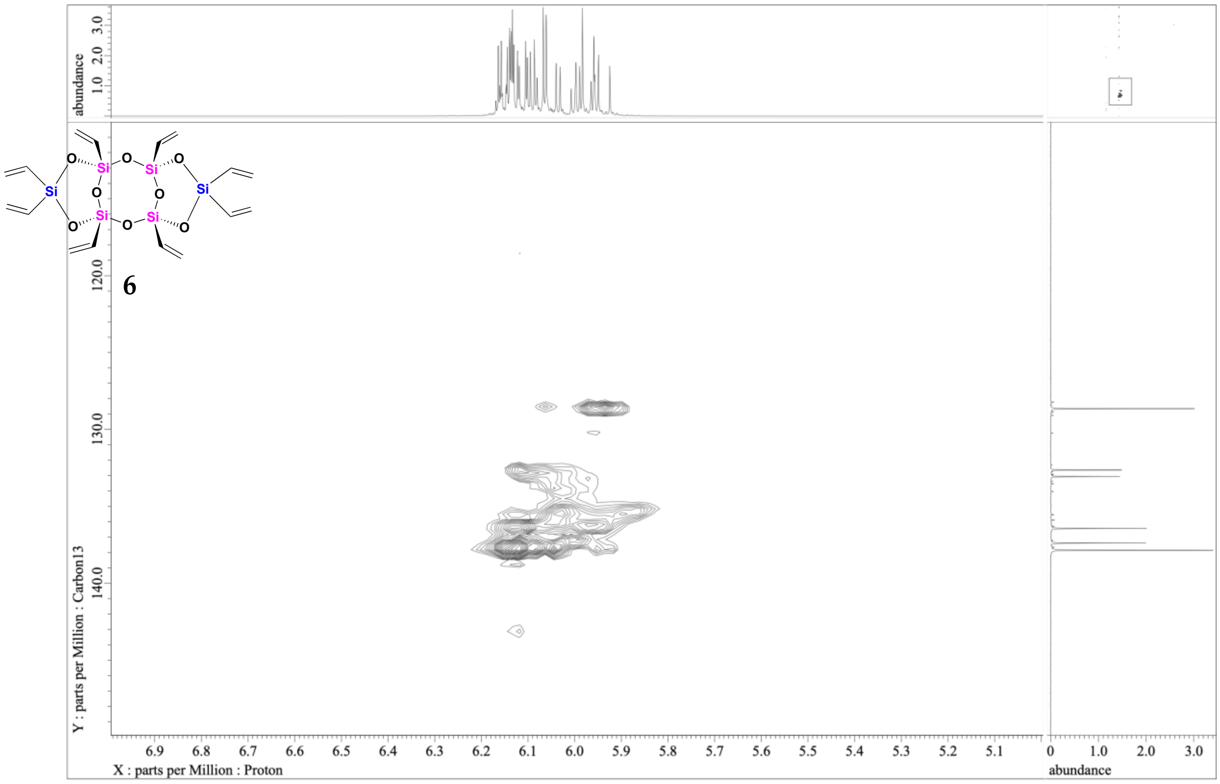

Figure S5. ^{1}H-^{13}C HSQC NMR spectrum for **6**.

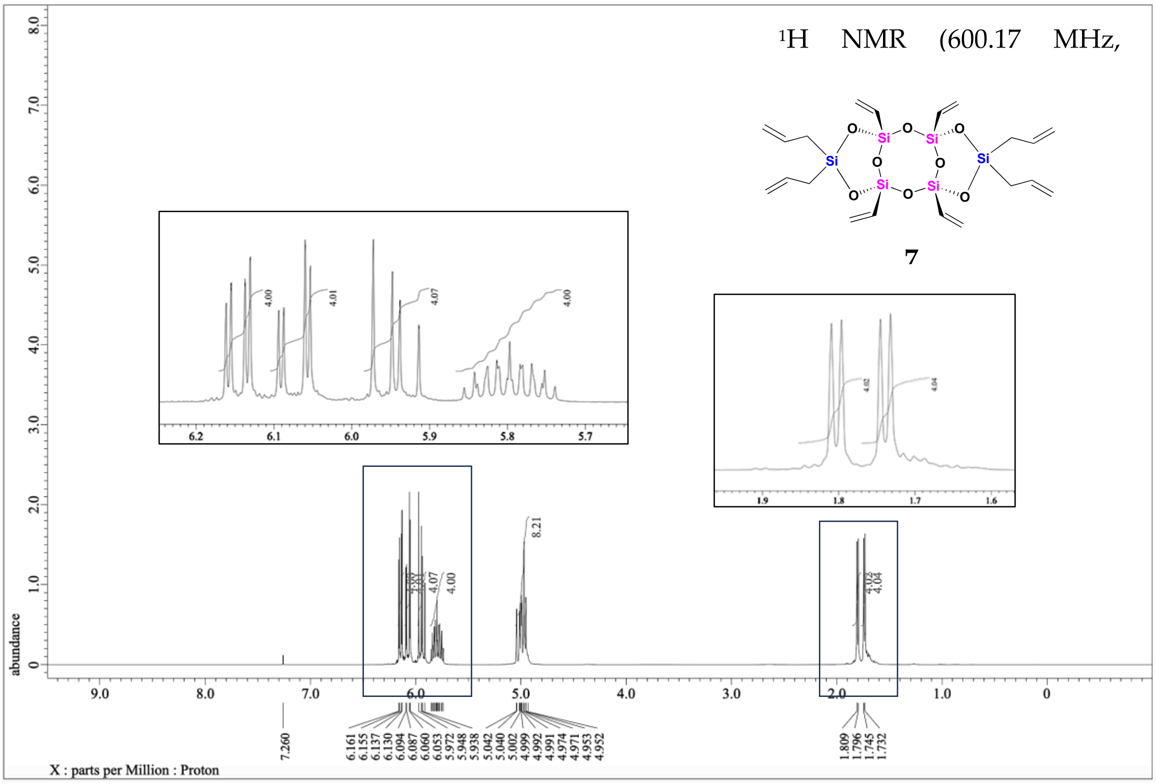

Figure S6. ^{1}H NMR spectrum for **7**.

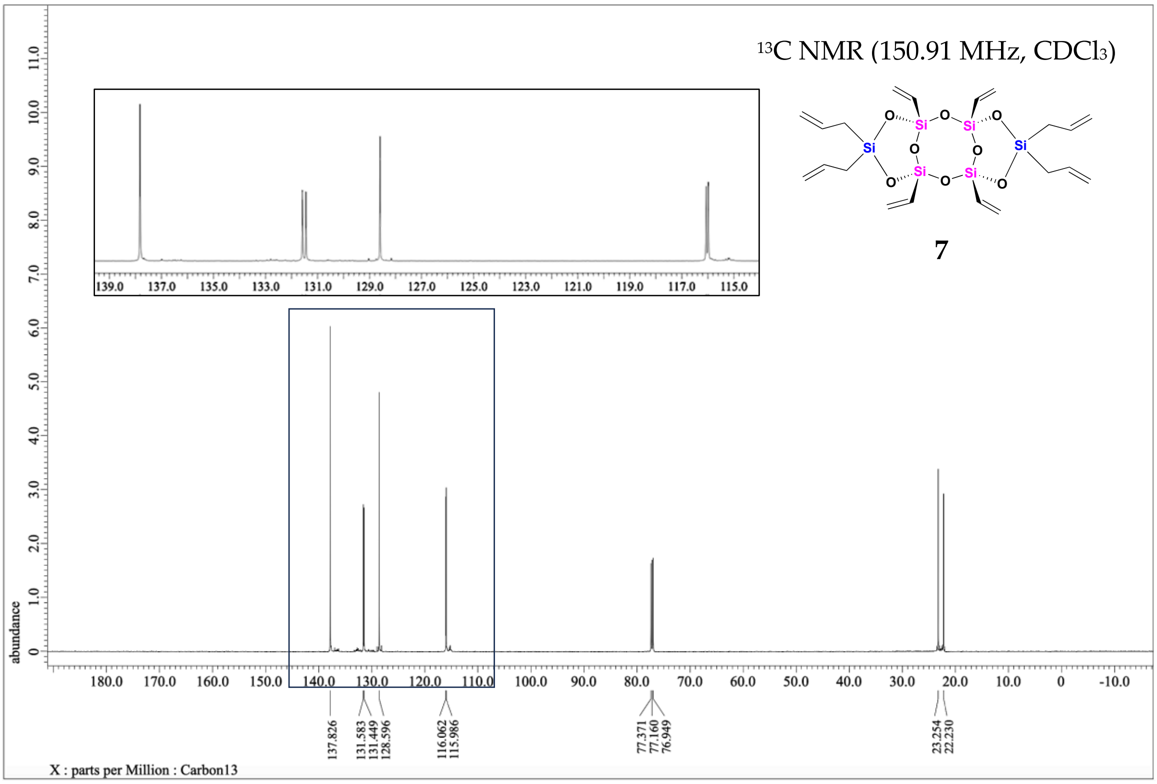

Figure S7. ^{13}C NMR spectrum for **7**.

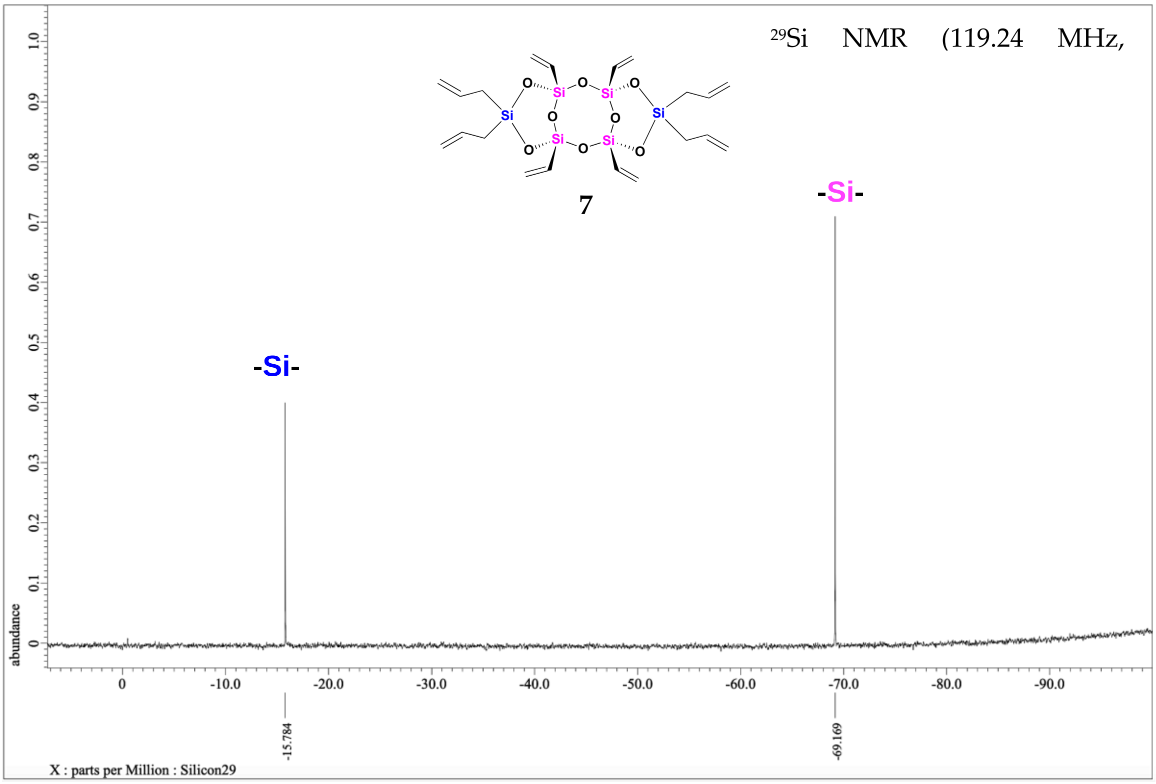

Figure S8. ^{29}Si NMR spectrum for **7**.

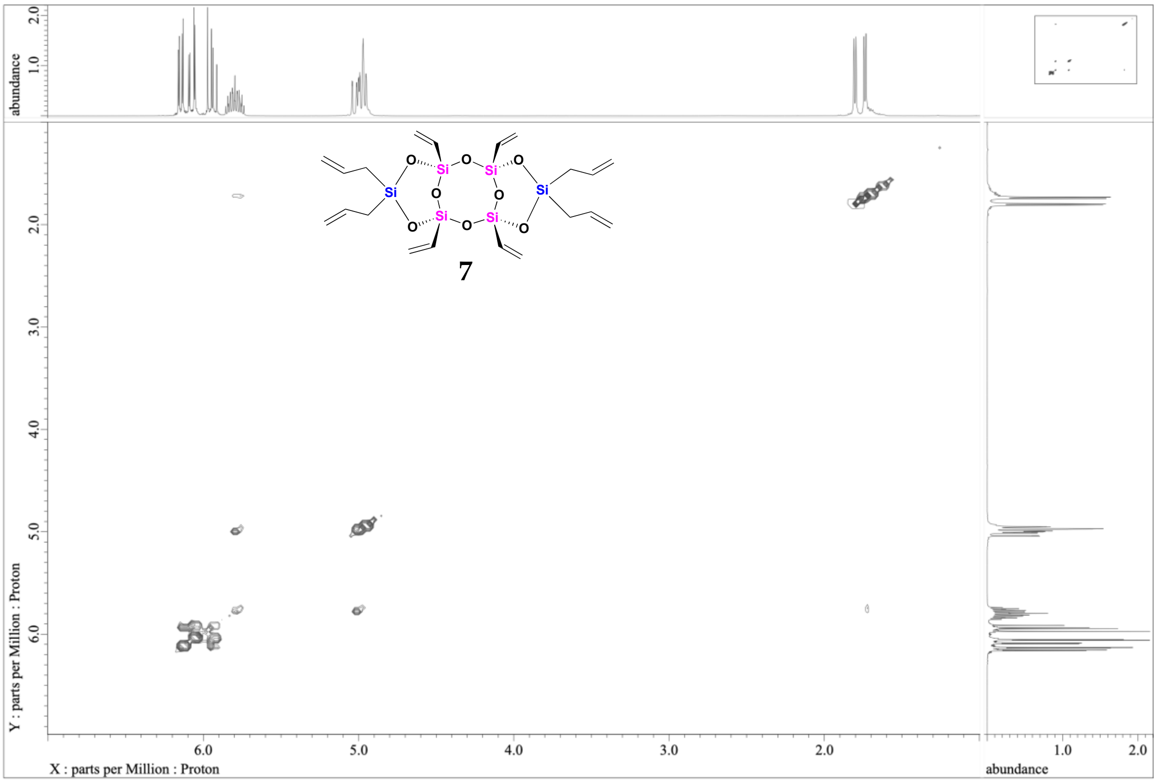

Figure S9. ^{1}H-^{1}H COSY NMR spectrum for **7**.

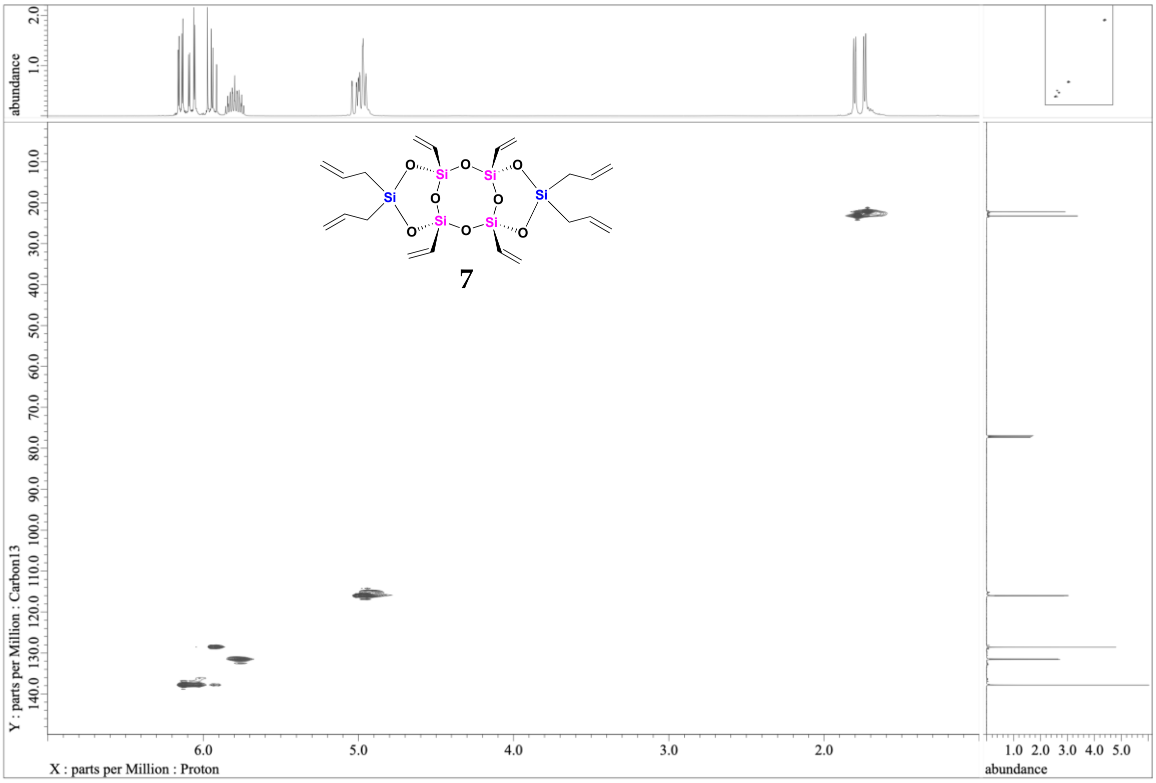

Figure S10. ^{1}H-^{13}C HSQC NMR spectrum for **7**.

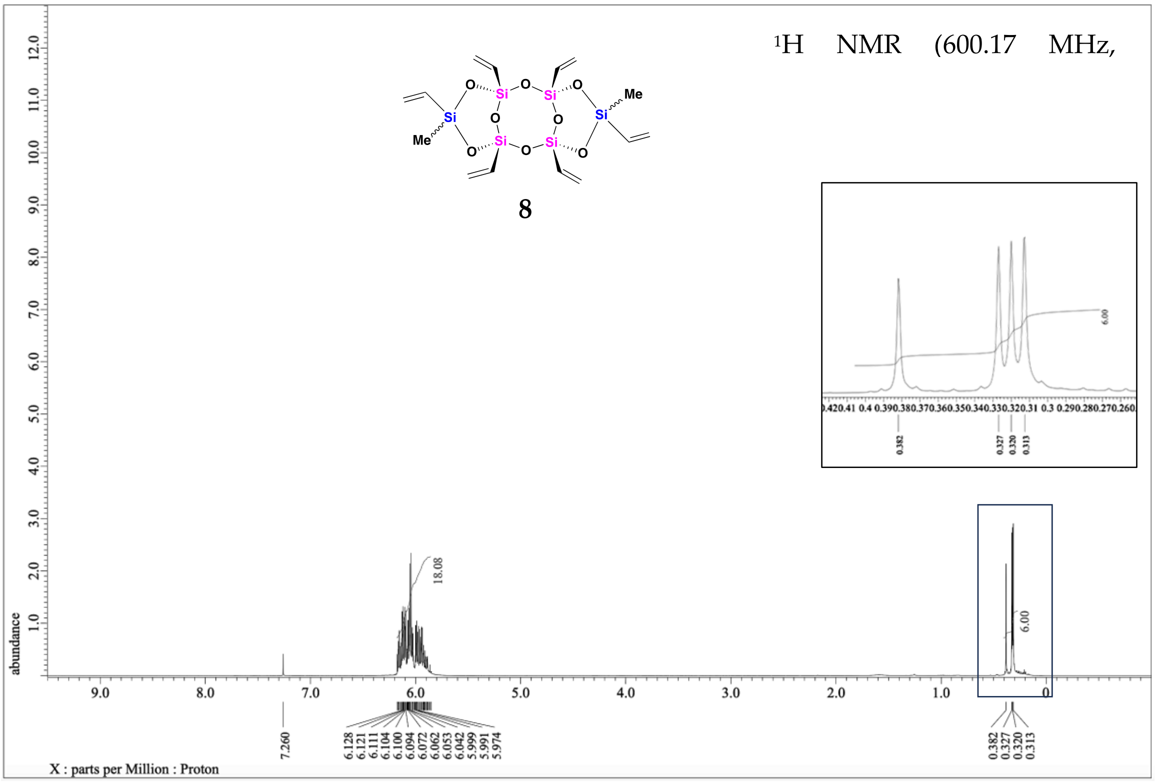

Figure S11. ^{1}H NMR spectrum for **8**.

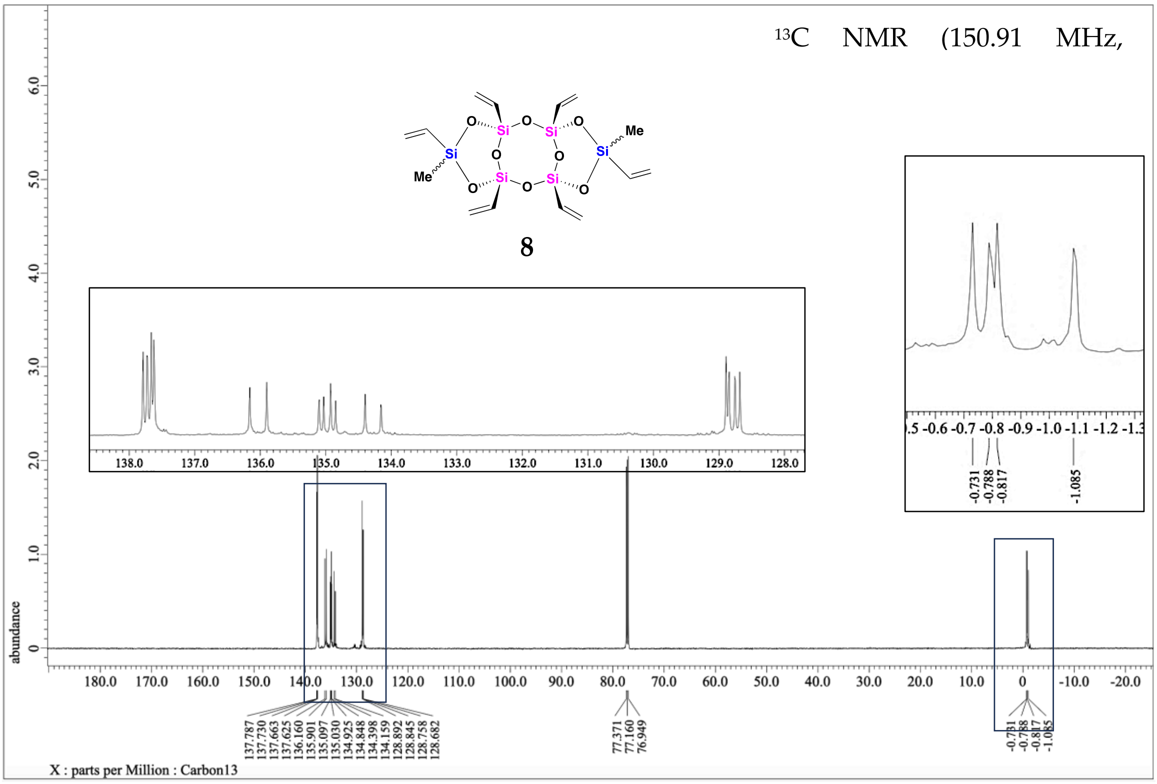

Figure S12. ^{13}C NMR spectrum for **8**.

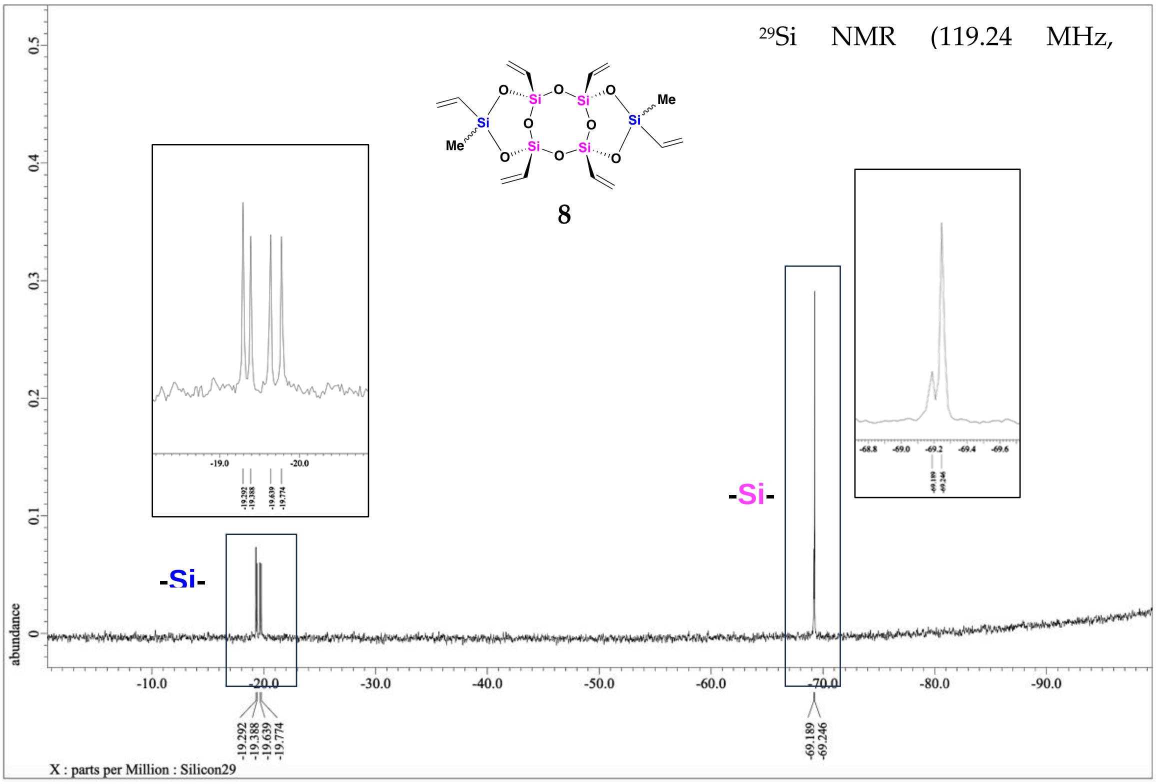

Figure S13. ^{29}Si NMR spectrum for **8**.

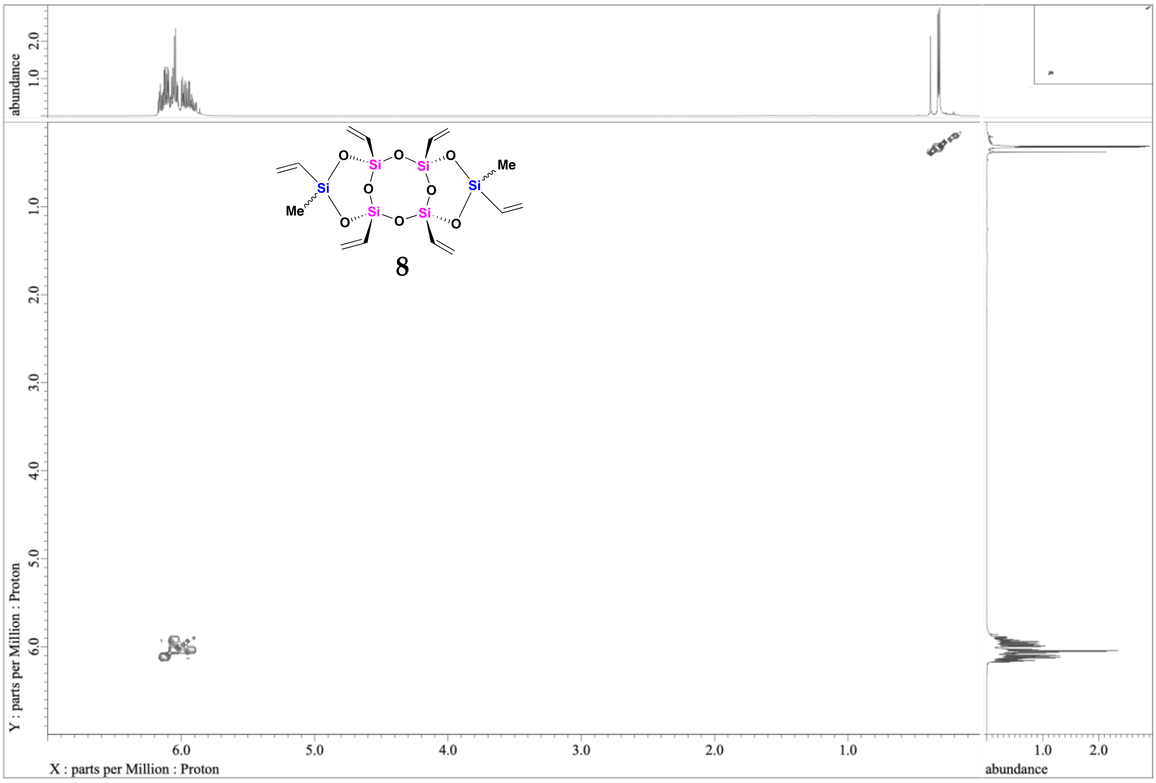

Figure S14. ^{1}H-^{1}H COSY NMR spectrum for **8**.

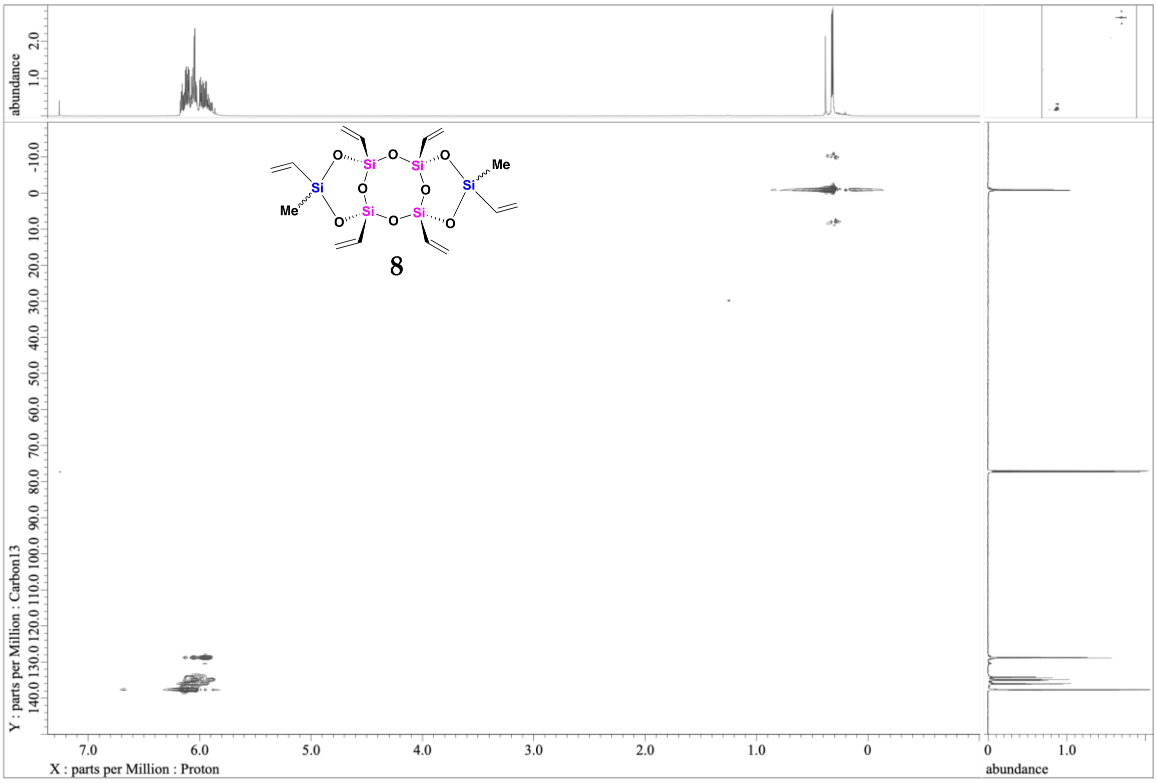

Figure S15. ^{1}H-^{13}C HSQC NMR spectrum for **8**.

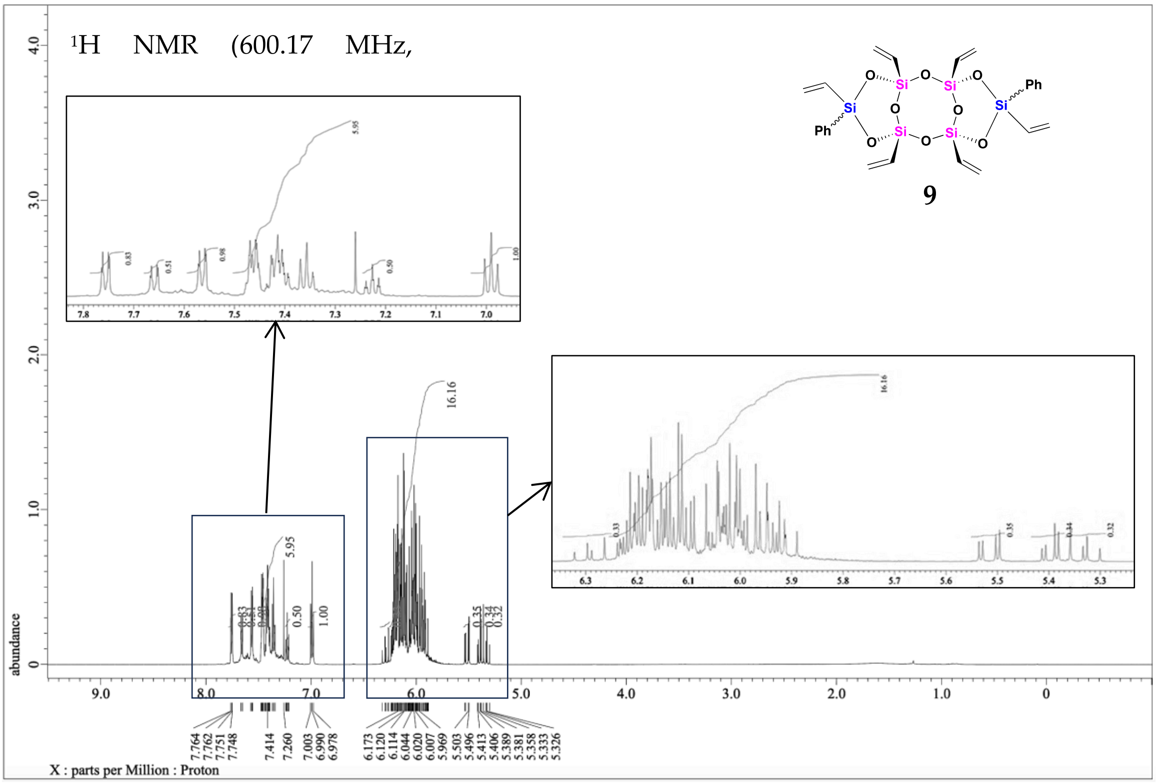

Figure S16. ^{1}H NMR spectrum for **9**.

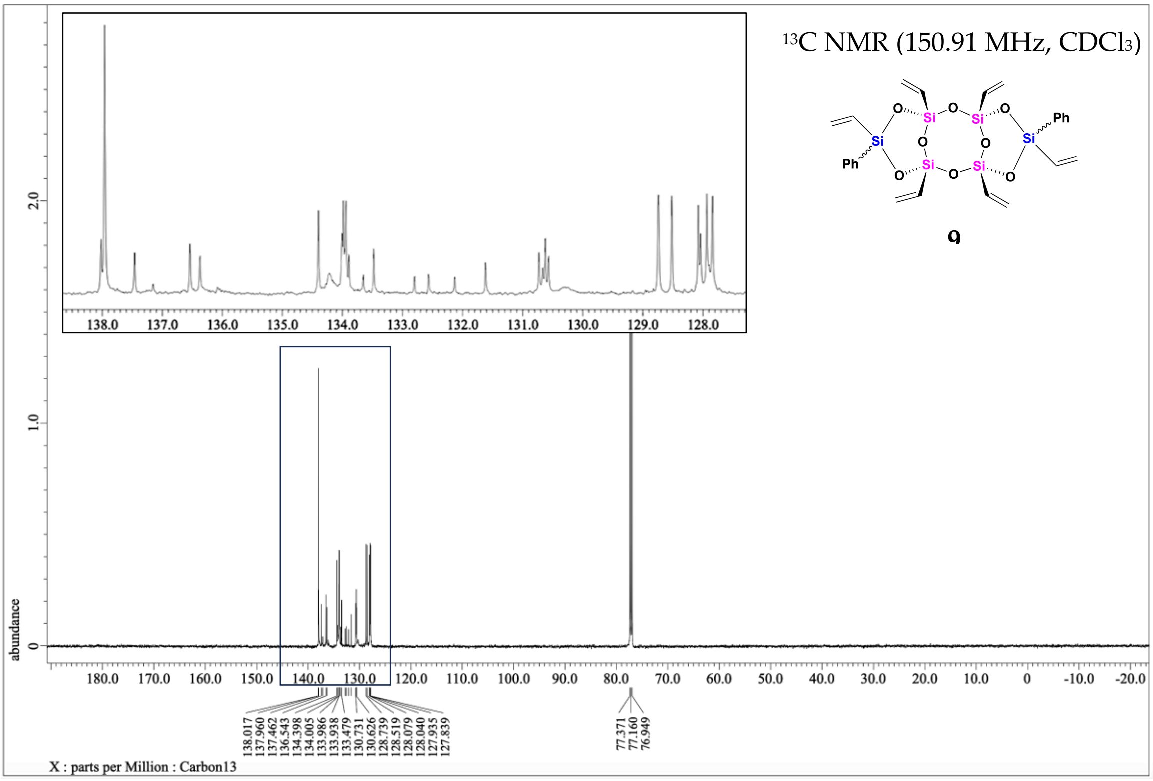

Figure S17. ^{13}C NMR spectrum for **9**.

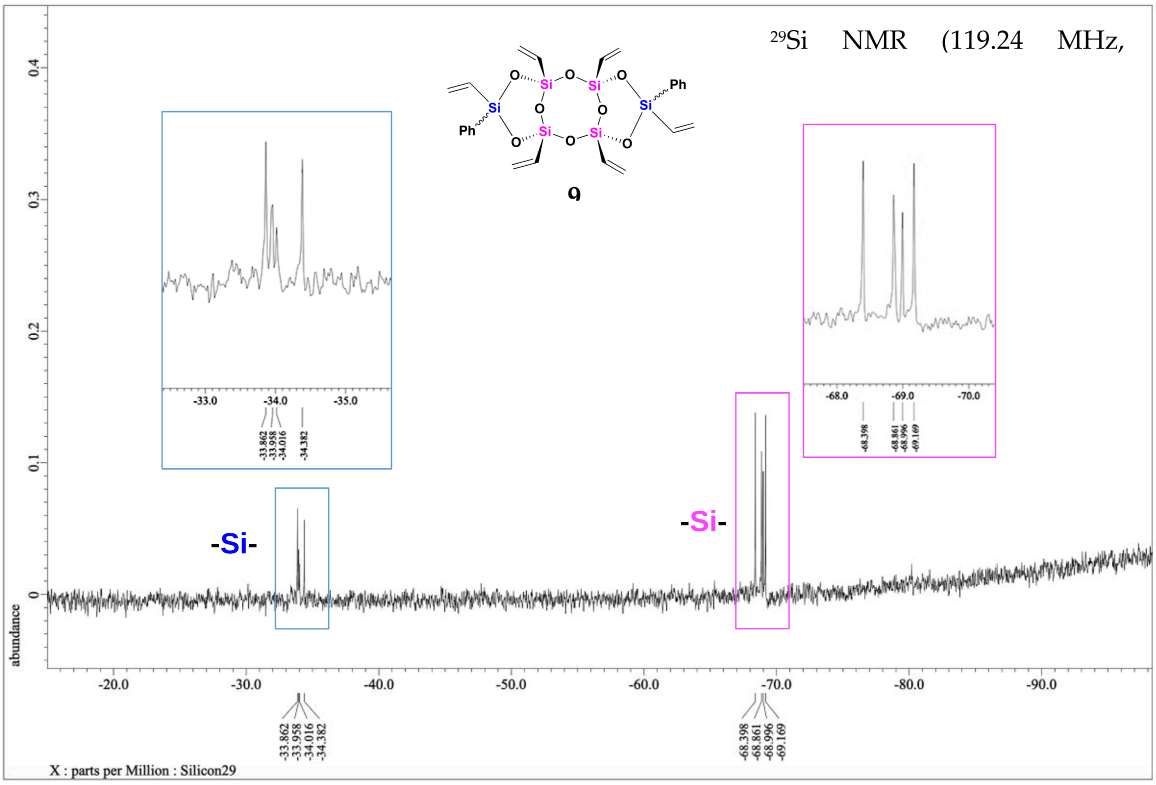

Figure S18. ^{29}Si NMR spectrum for **9**.

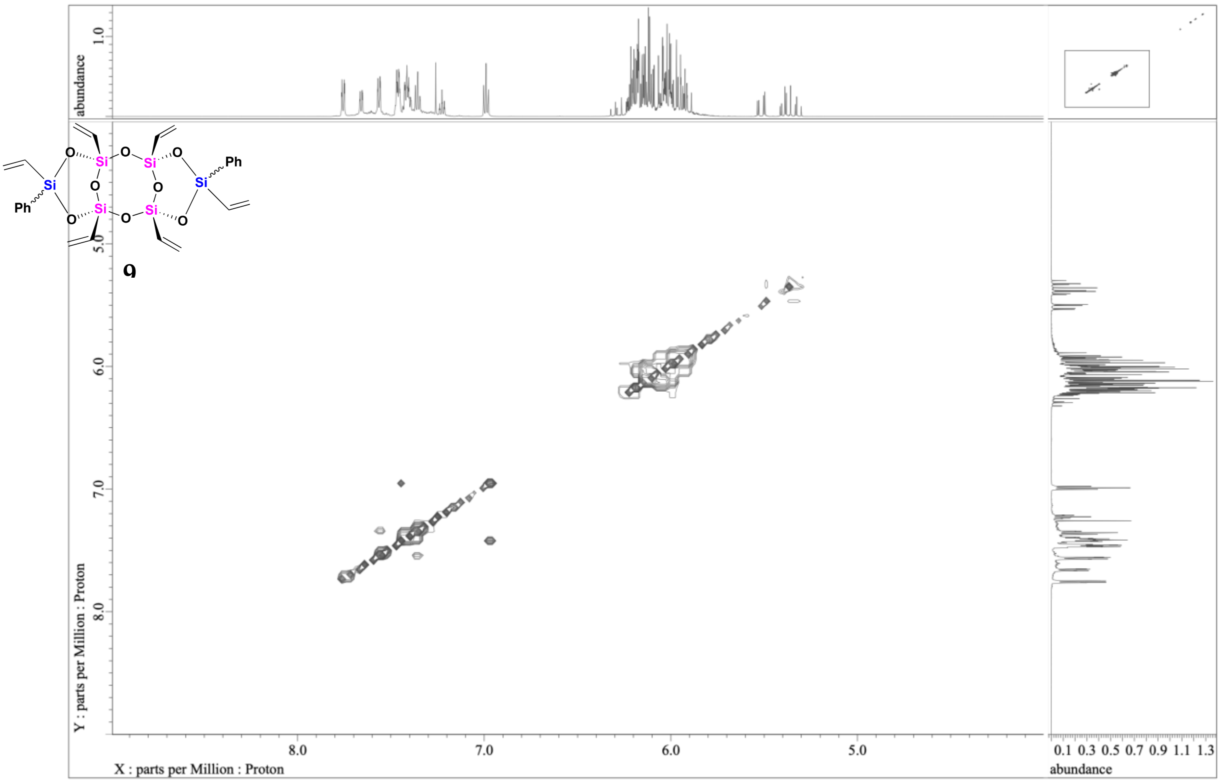

Figure S19. ^1H-^1H COSY NMR spectrum for **9**.

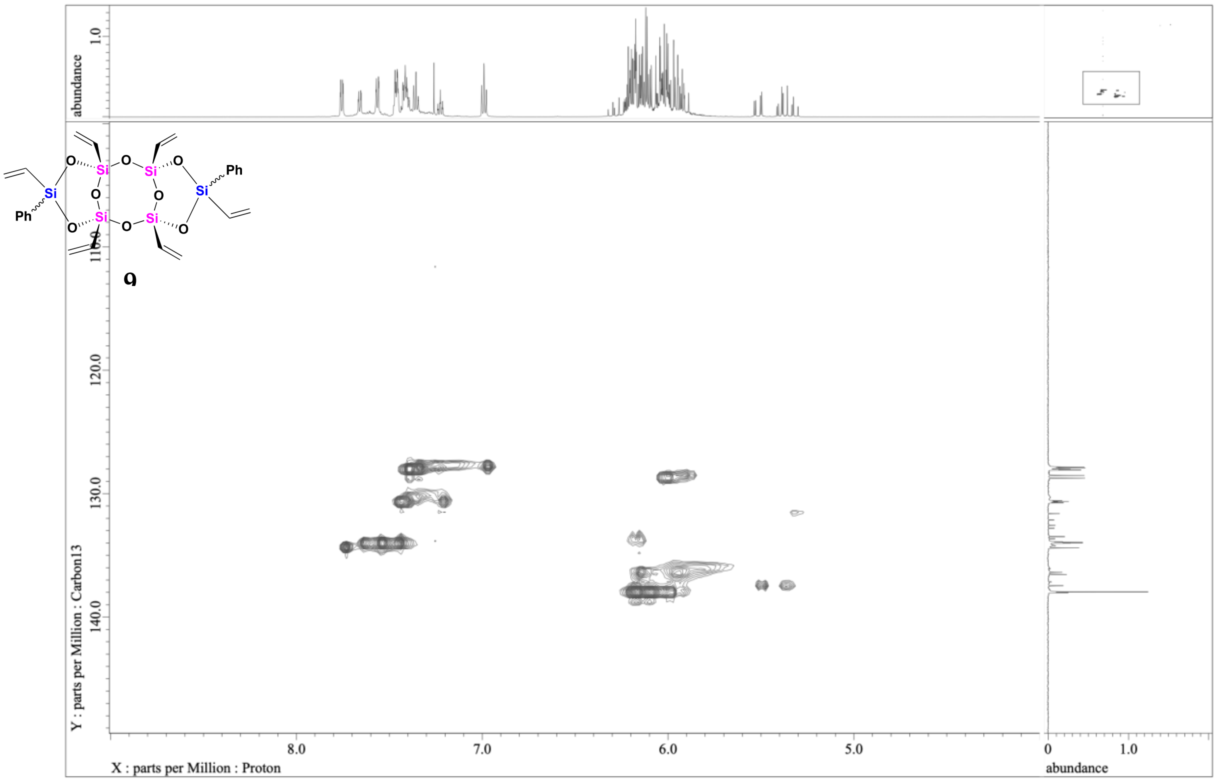

Figure S20. ^1H-^{13}C HSQC NMR spectrum for **9**.

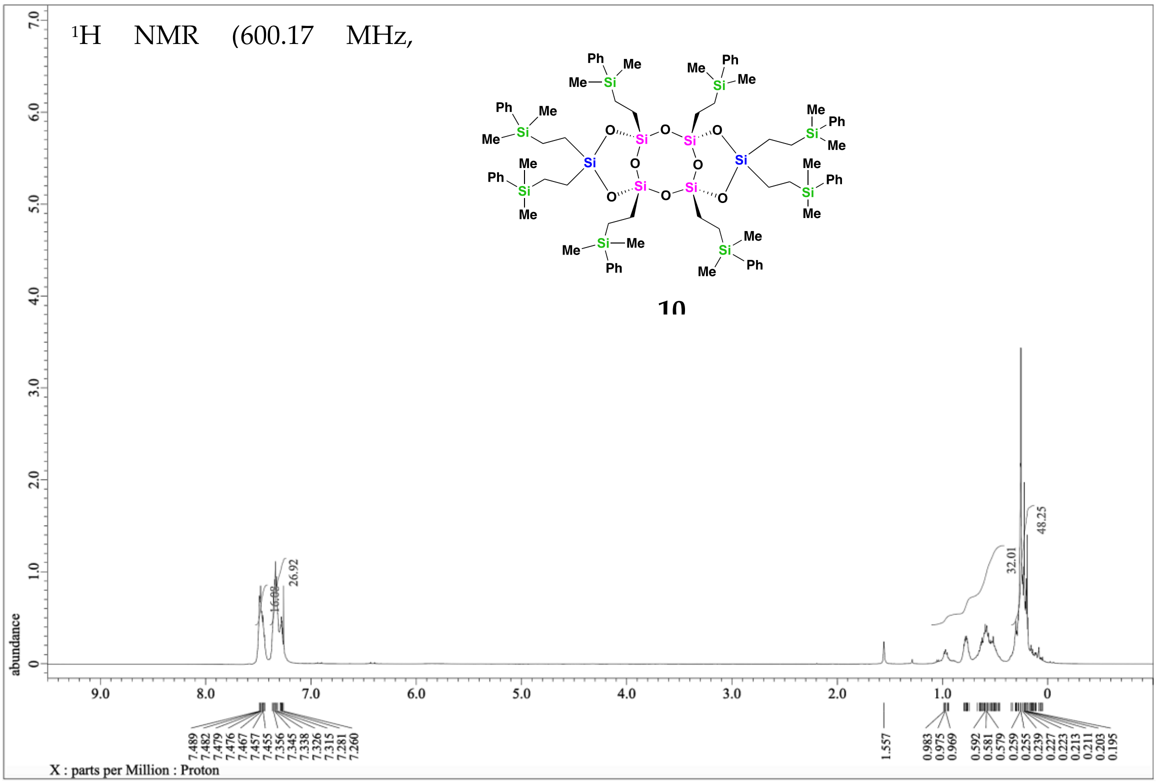

Figure S21. ^{1}H NMR spectrum for **10**.

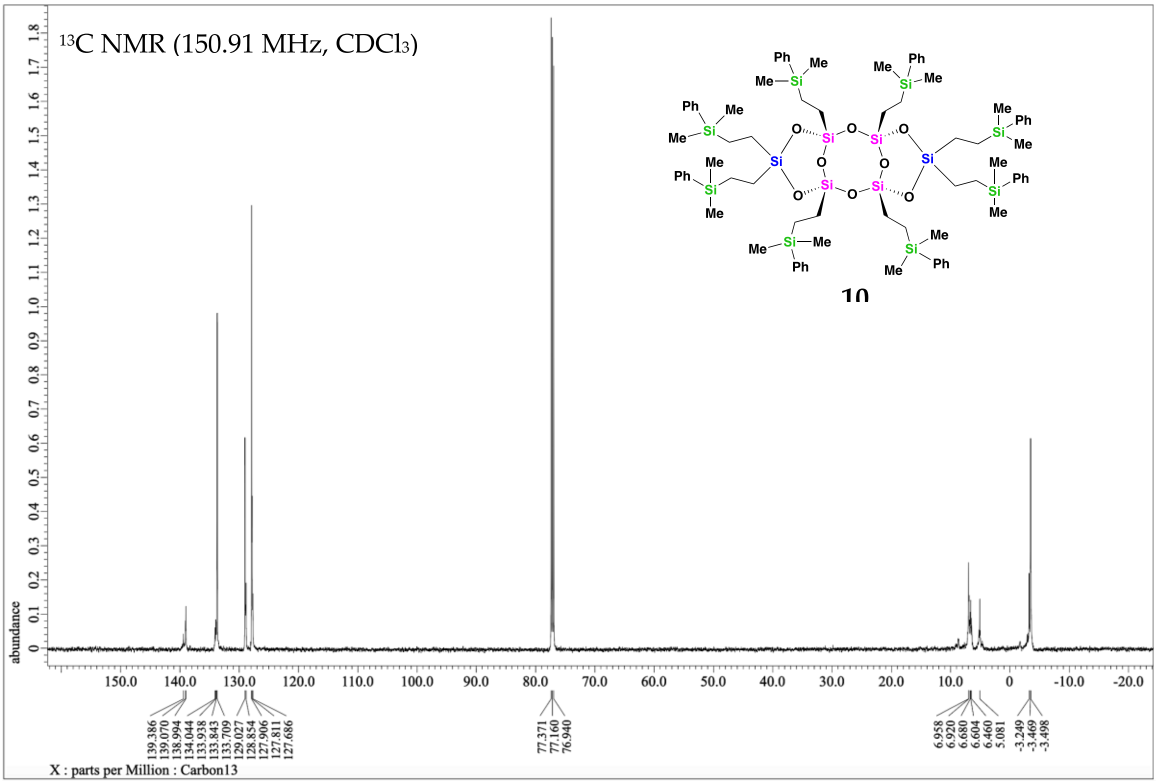

Figure S22. ^{13}C NMR spectrum for **10**.

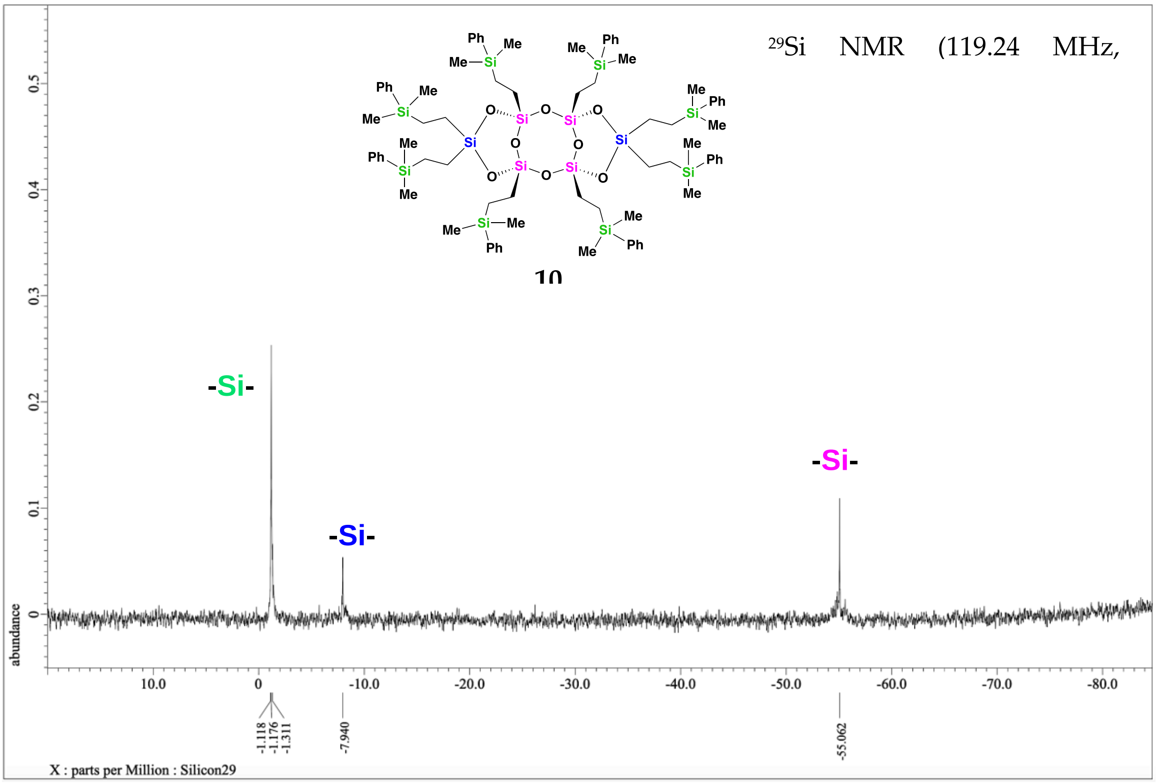

Figure S23. ^{29}Si NMR spectrum for **10**.

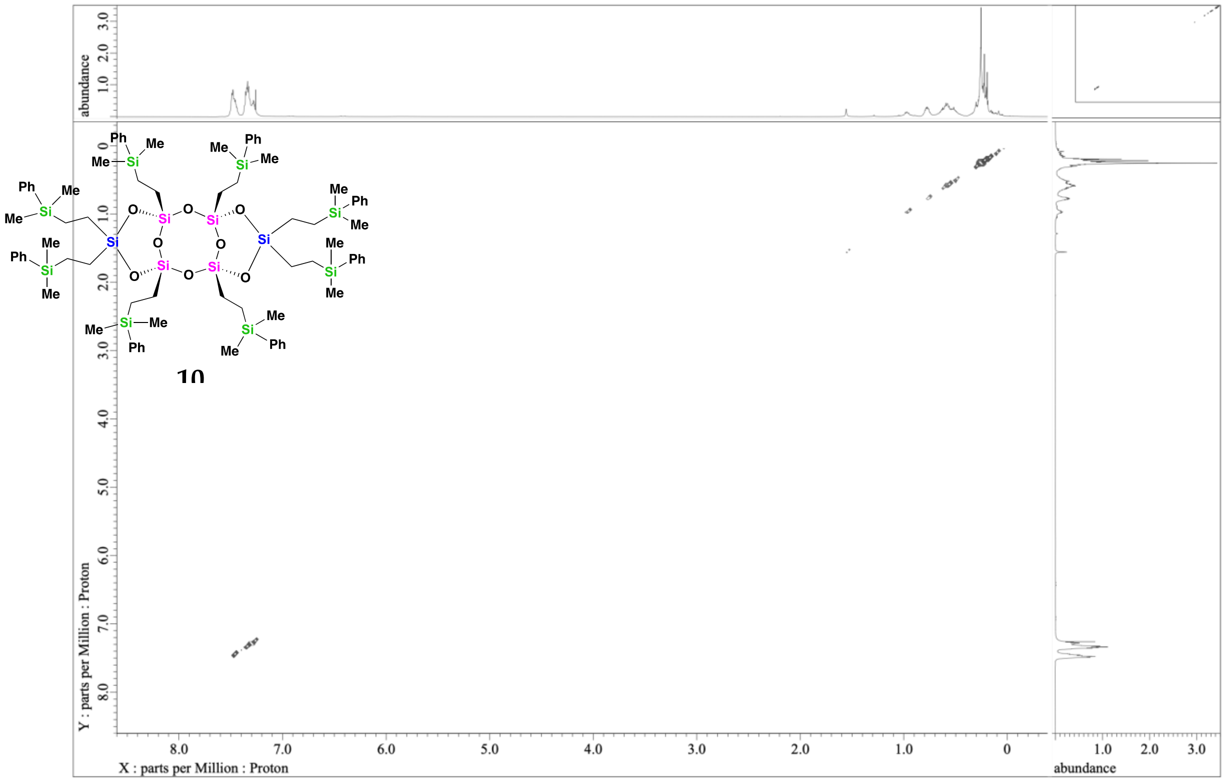

Figure S24. ^{1}H-^{1}H COSY NMR spectrum for **10**.

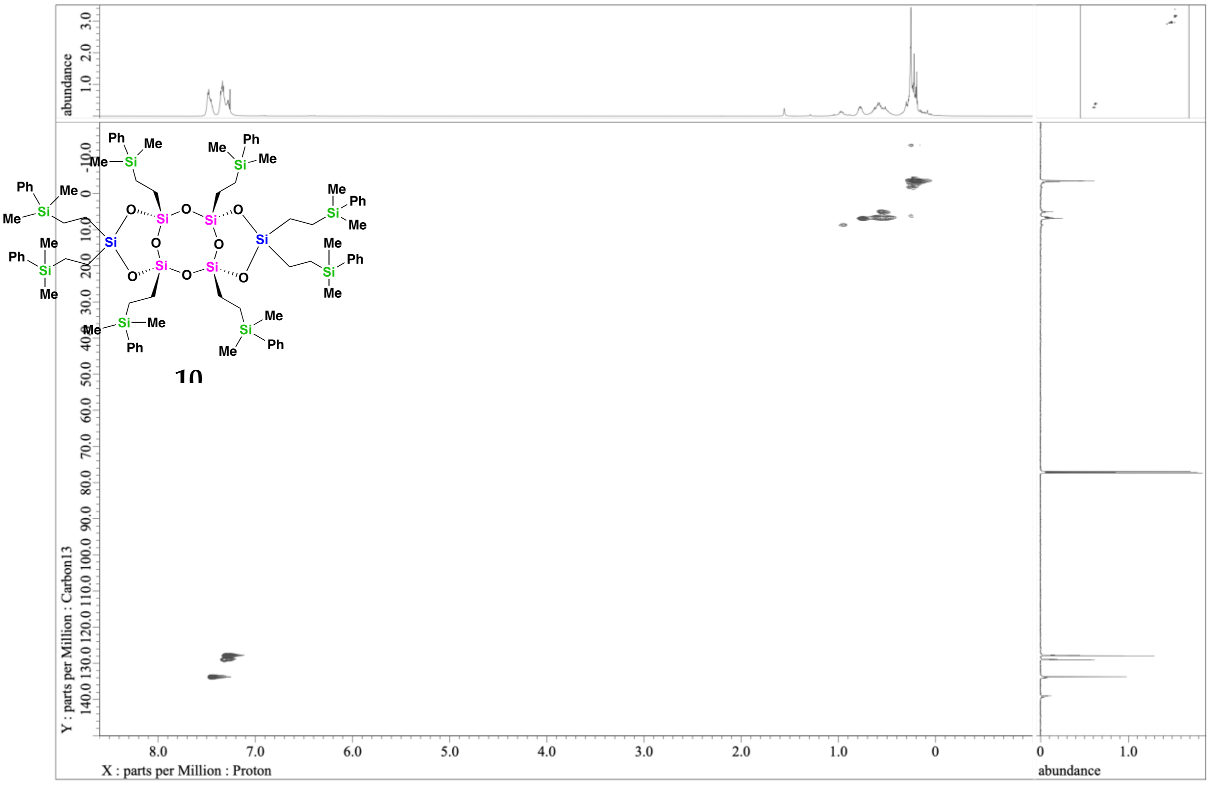

Figure S25. ^{1}H-^{13}C HSQC NMR spectrum for **10**.

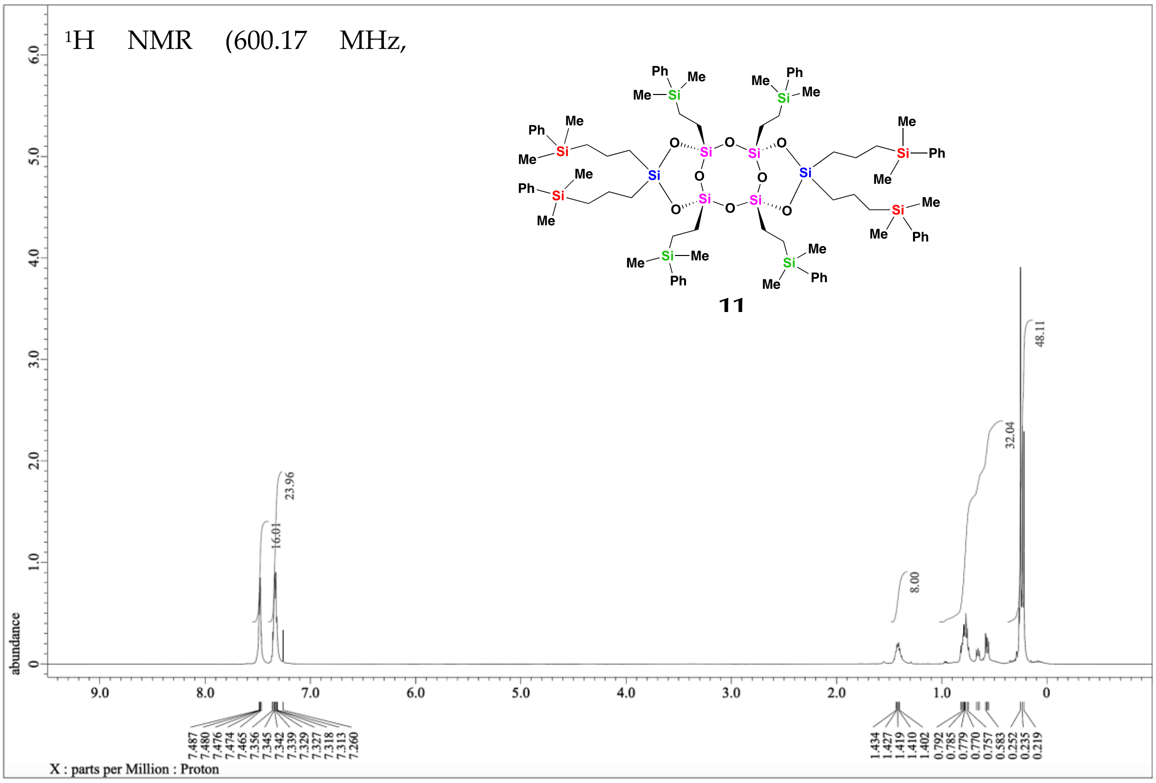

Figure S26. ^{1}H NMR spectrum for **11**.

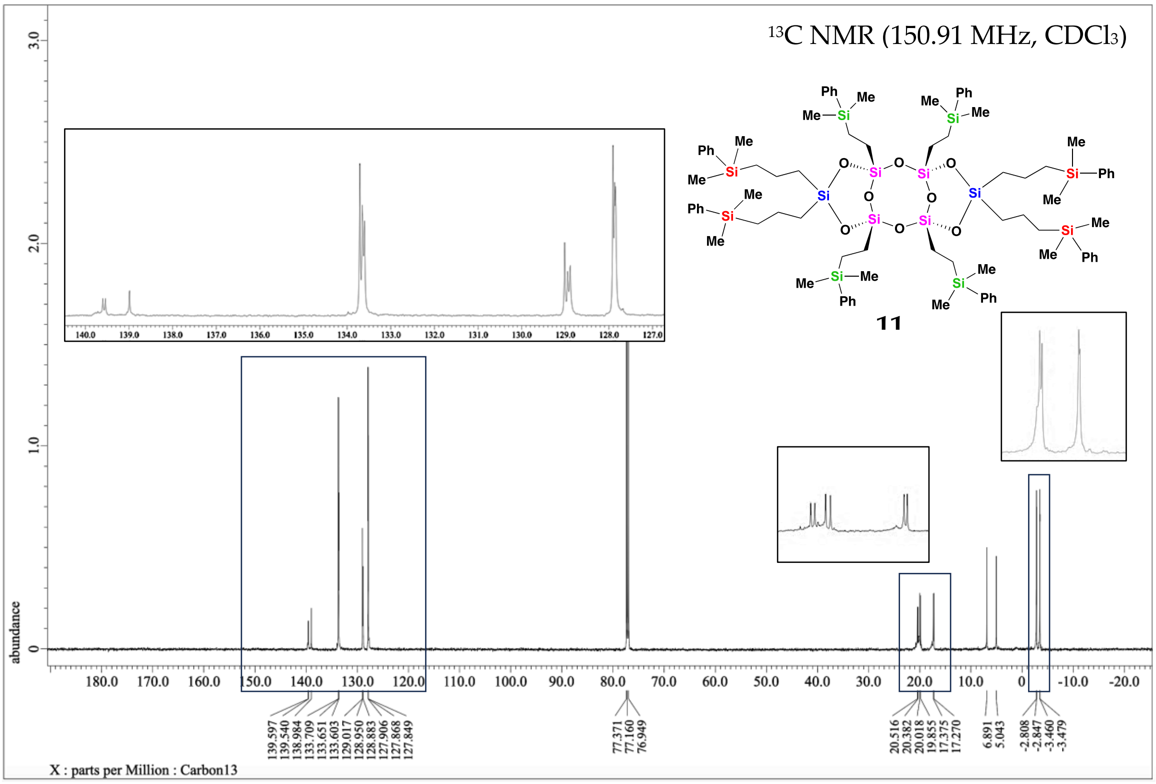

Figure S27. ^{13}C NMR spectrum for **11**.

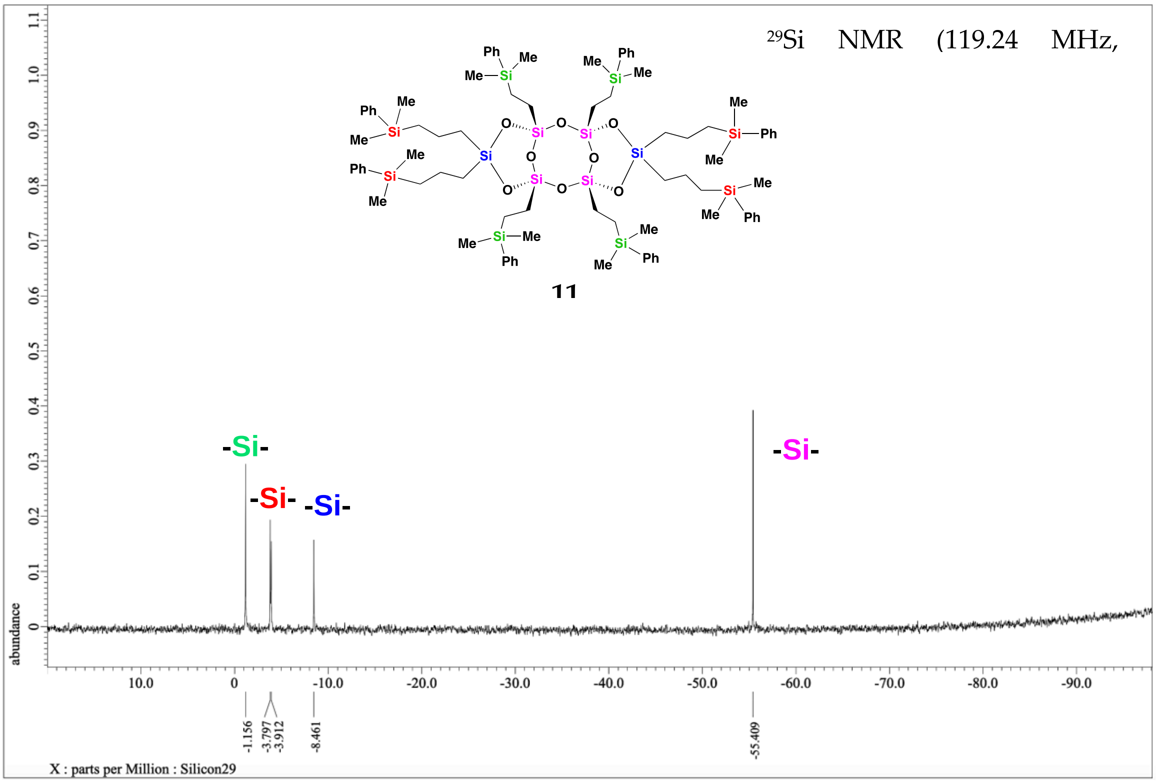

Figure S28. ^{29}Si NMR spectrum for **11**.

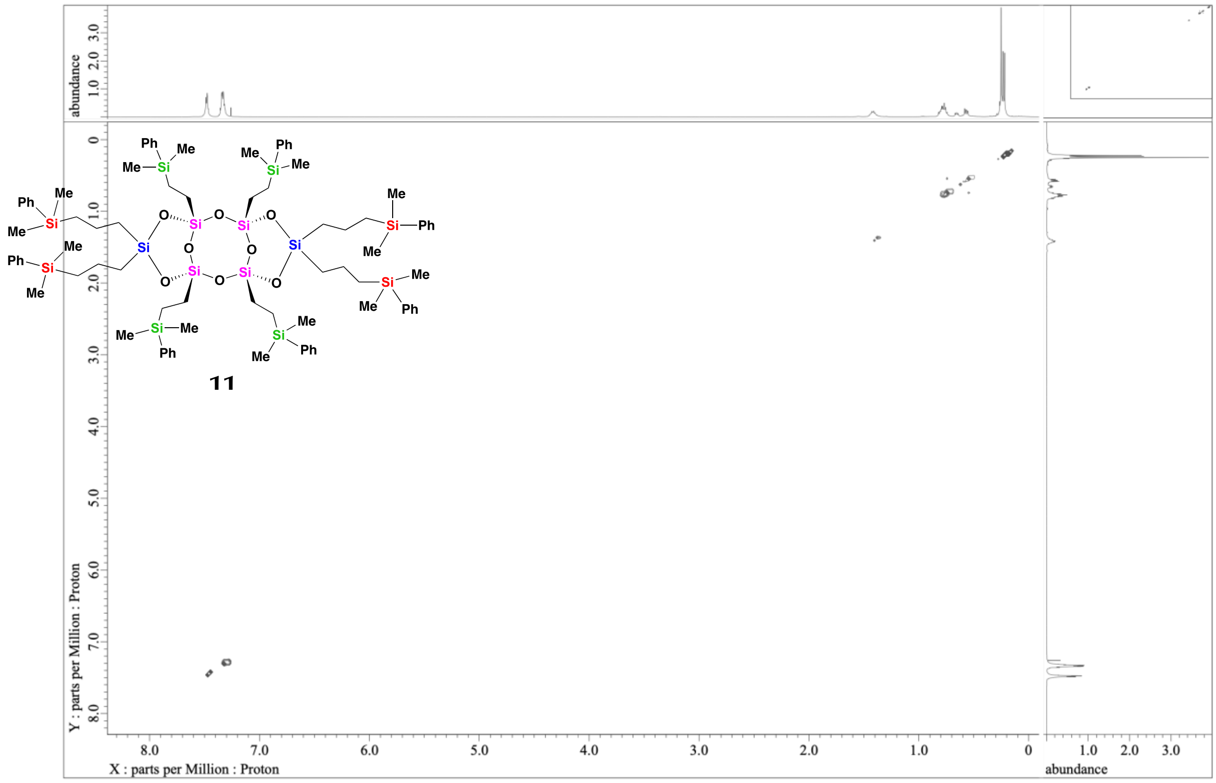

Figure S29. ^{1}H-^{1}H COSY NMR spectrum for **11**.

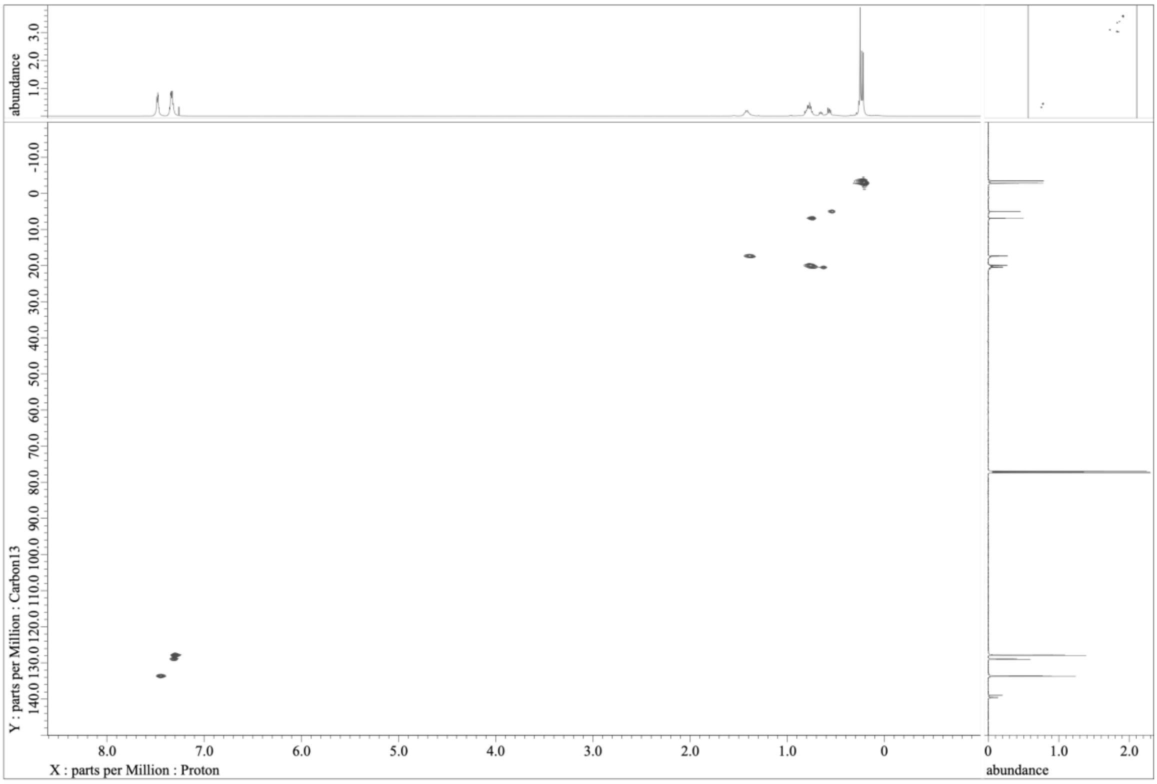

Figure S30. ^{1}H-^{13}C HSQC NMR spectrum for **11**.

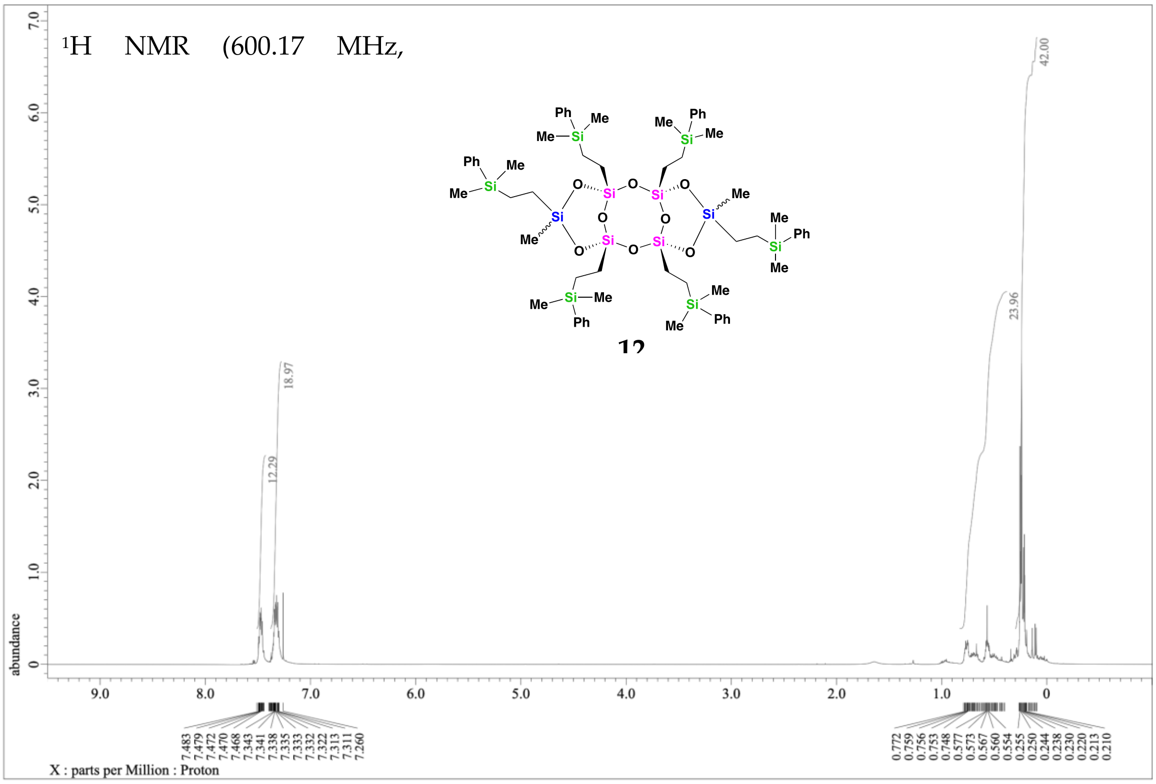

Figure S31. ^{1}H NMR spectrum for **12**.

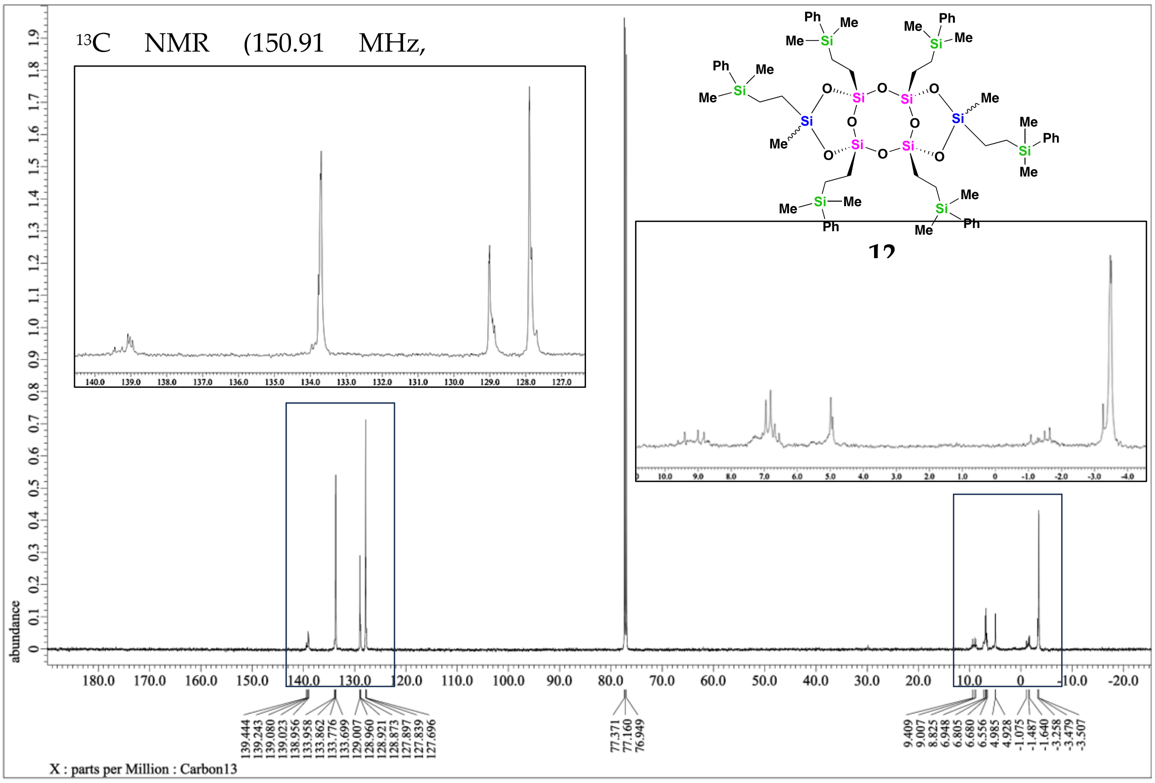

Figure S32. ^{13}C NMR spectrum for **12**.

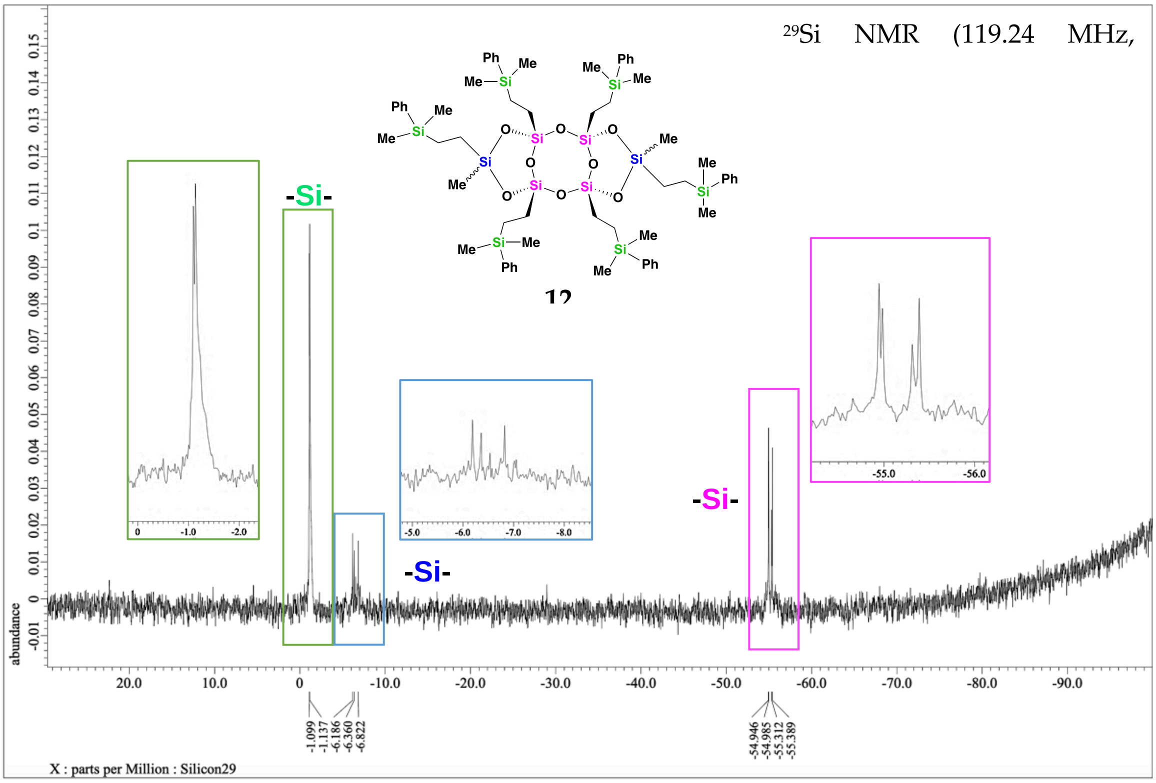

Figure S33. ^{29}Si NMR spectrum for **12**.

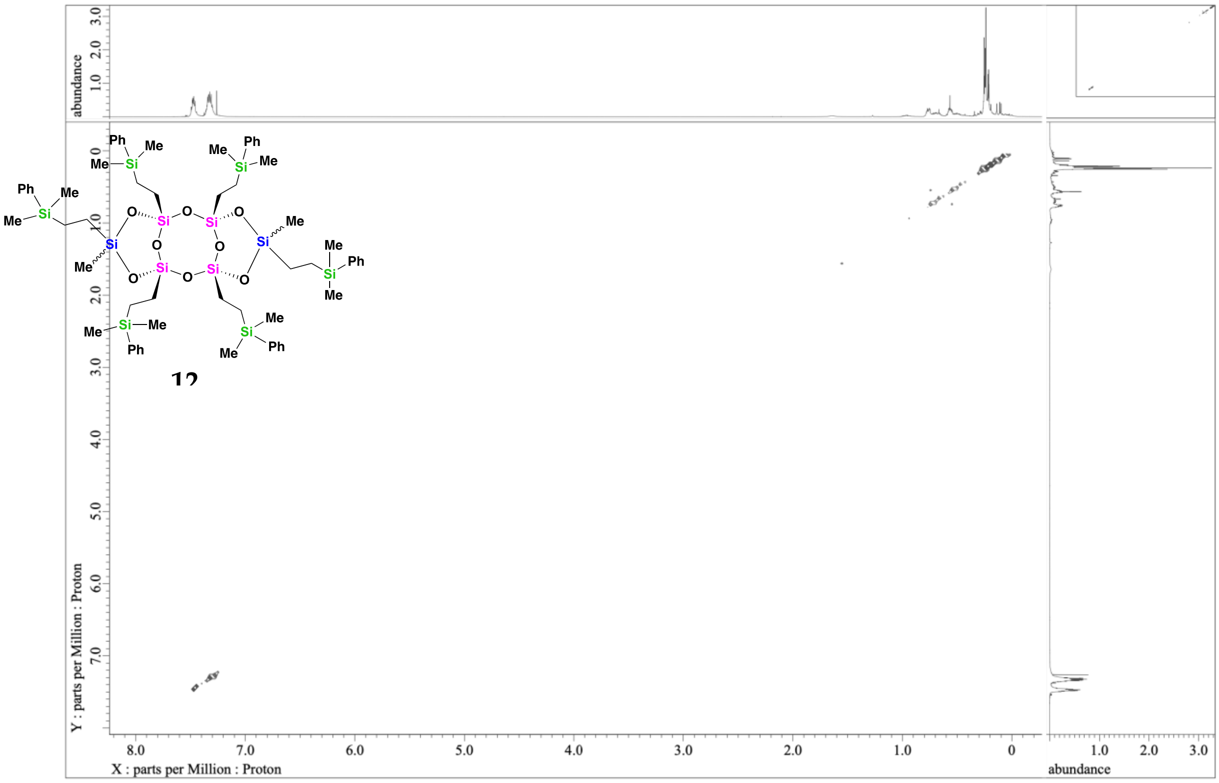

Figure S34. ^{1}H-^{1}H COSY NMR spectrum for **12**.

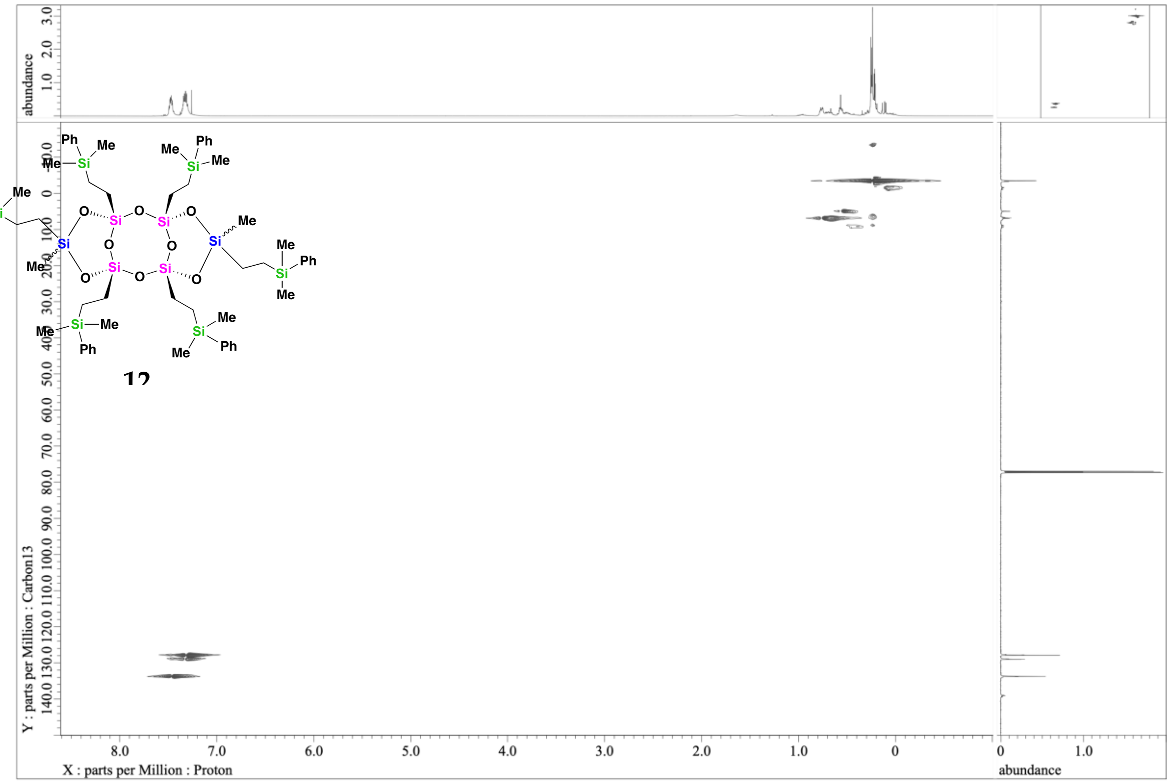

Figure S35. ^{1}H-^{13}C HSQC NMR spectrum for **12**.

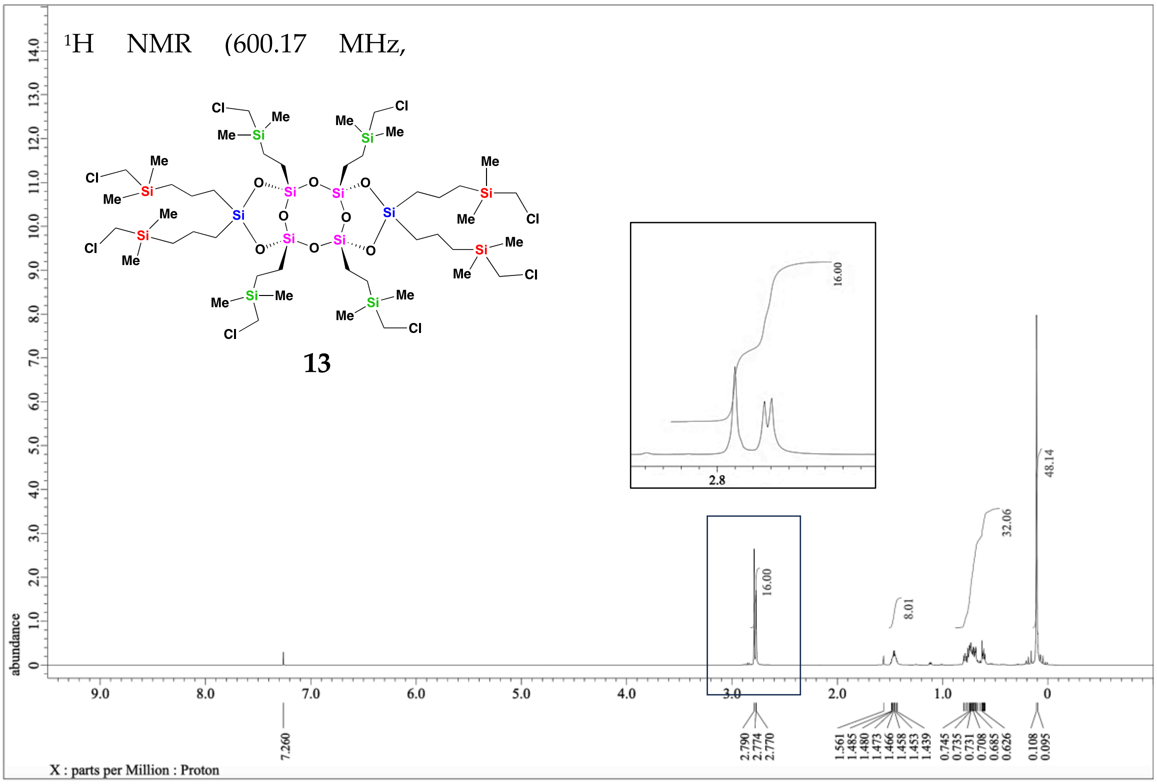

Figure S36. ^{1}H NMR spectrum for **13**.

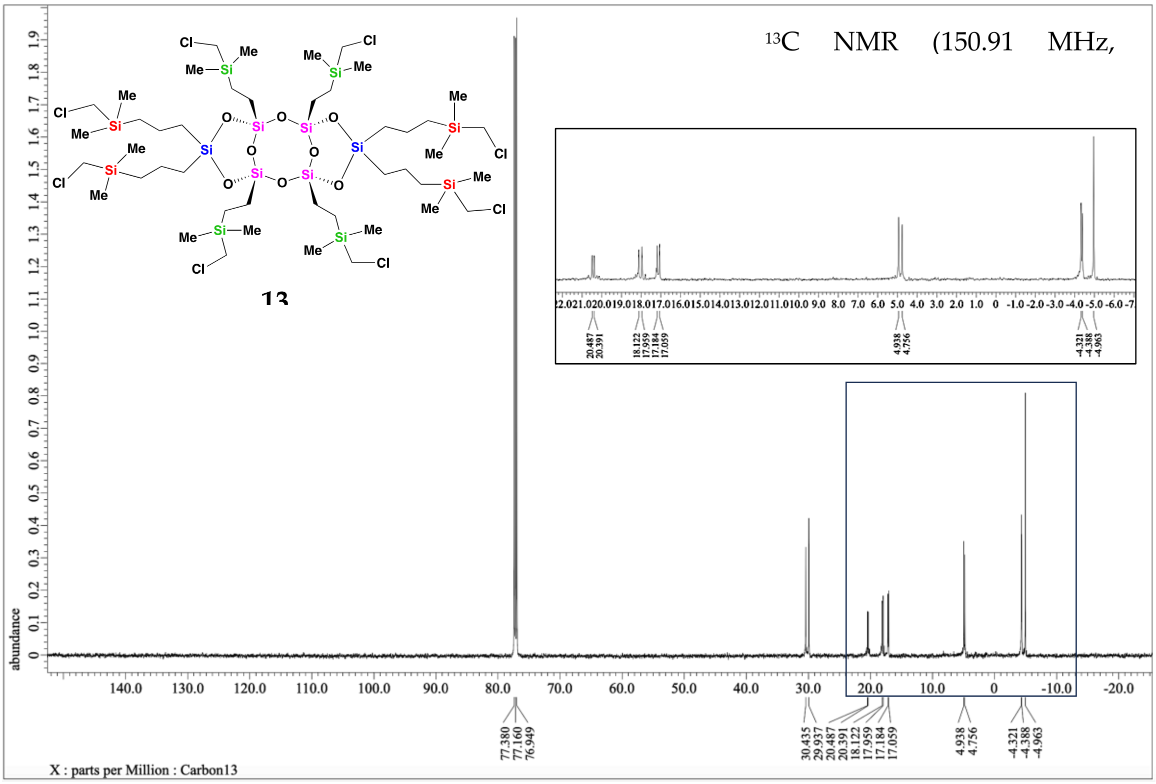

Figure S37. ^{13}C NMR spectrum for **13**.

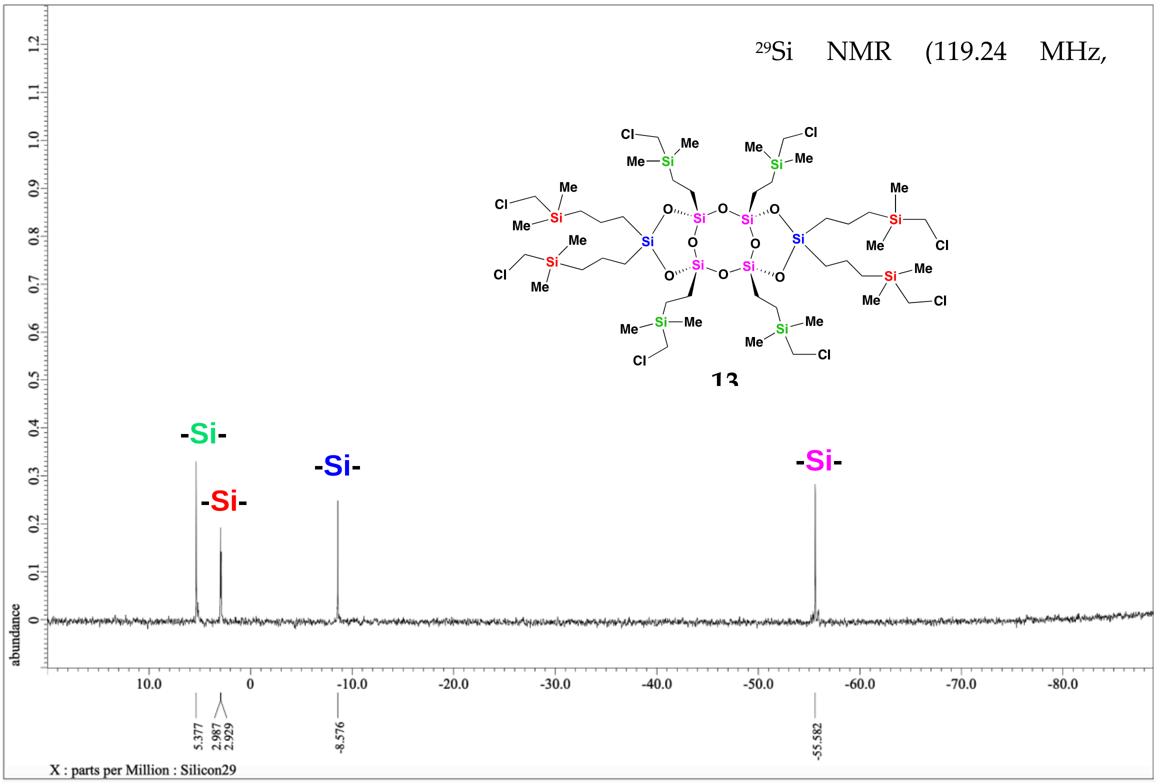

Figure S38. ^{29}Si NMR spectrum for **13**.

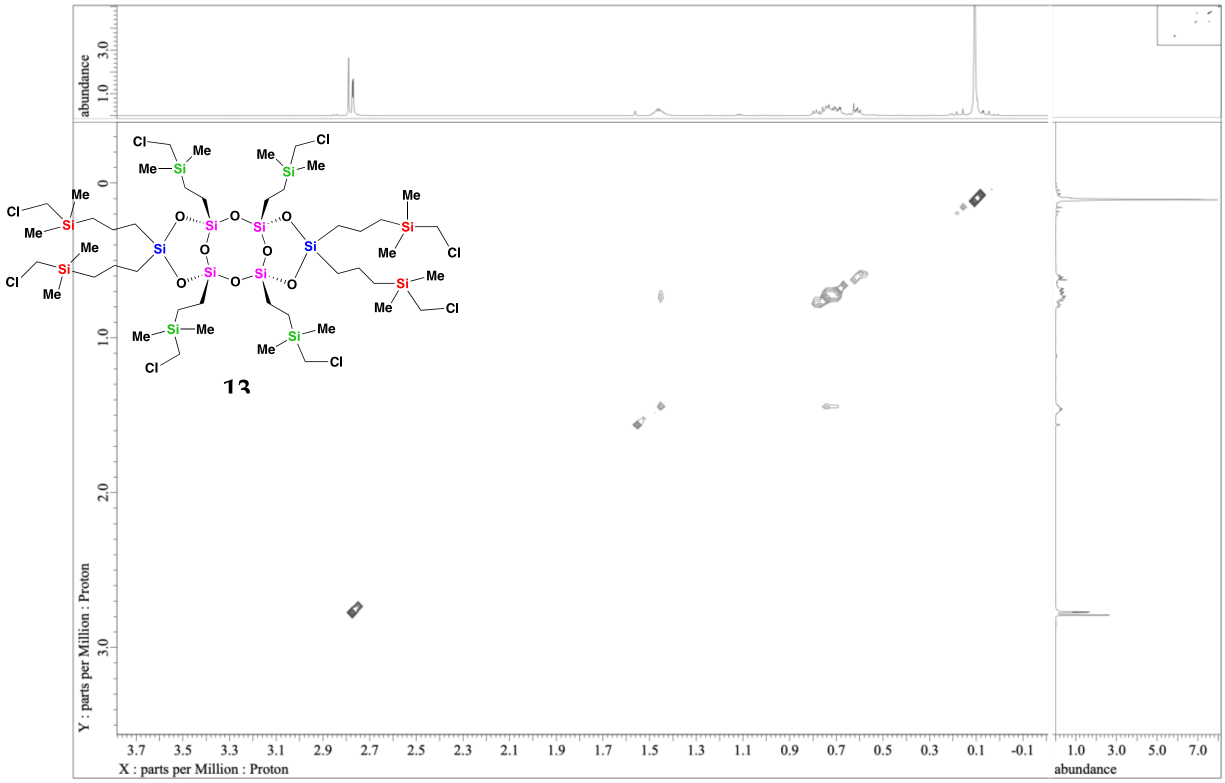

Figure S39. ^{1}H-^{1}H COSY NMR spectrum for **13**.

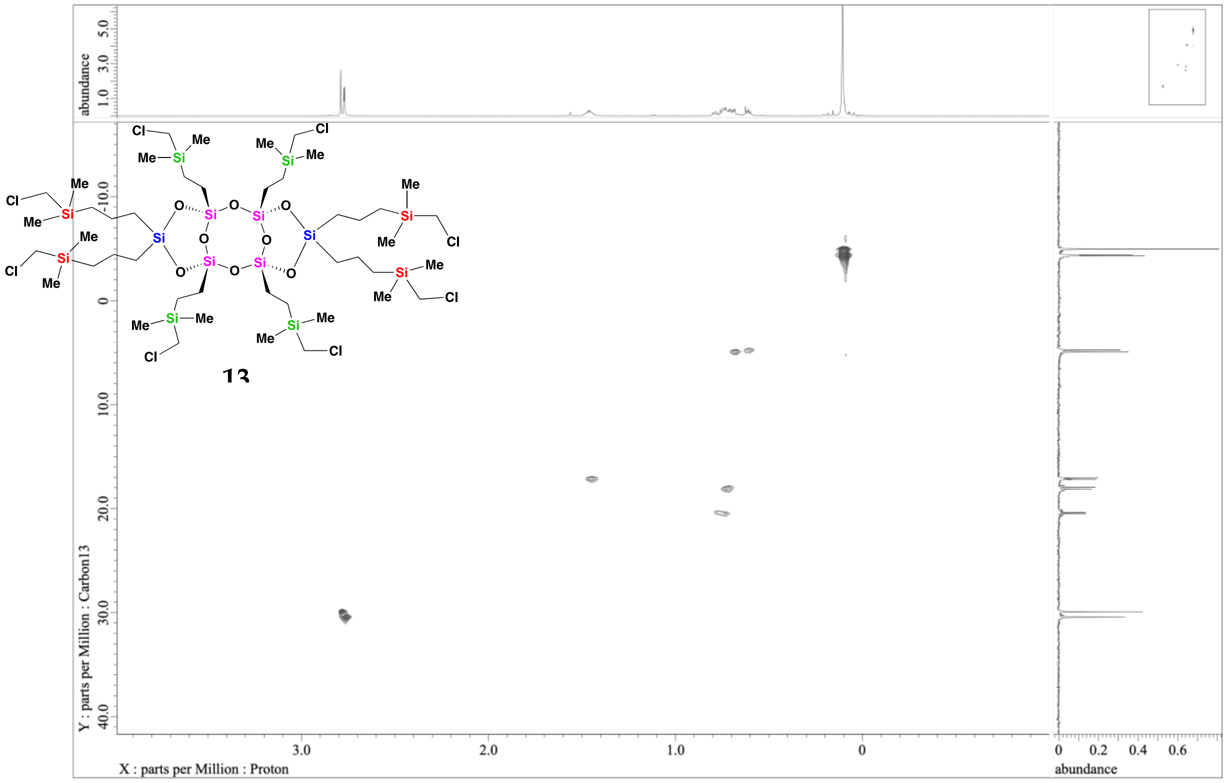

Figure S40. ^{1}H-^{13}C HSQC NMR spectrum for **13**.

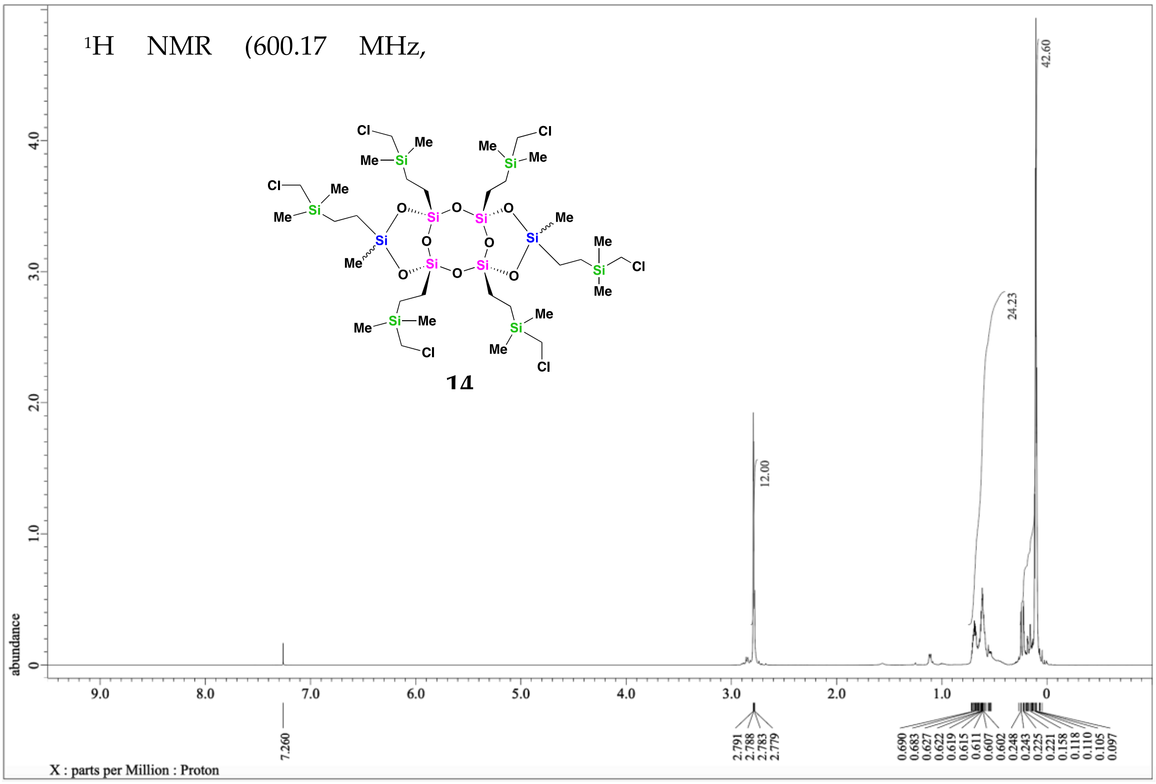

Figure S41. ^{1}H NMR spectrum for **14**.

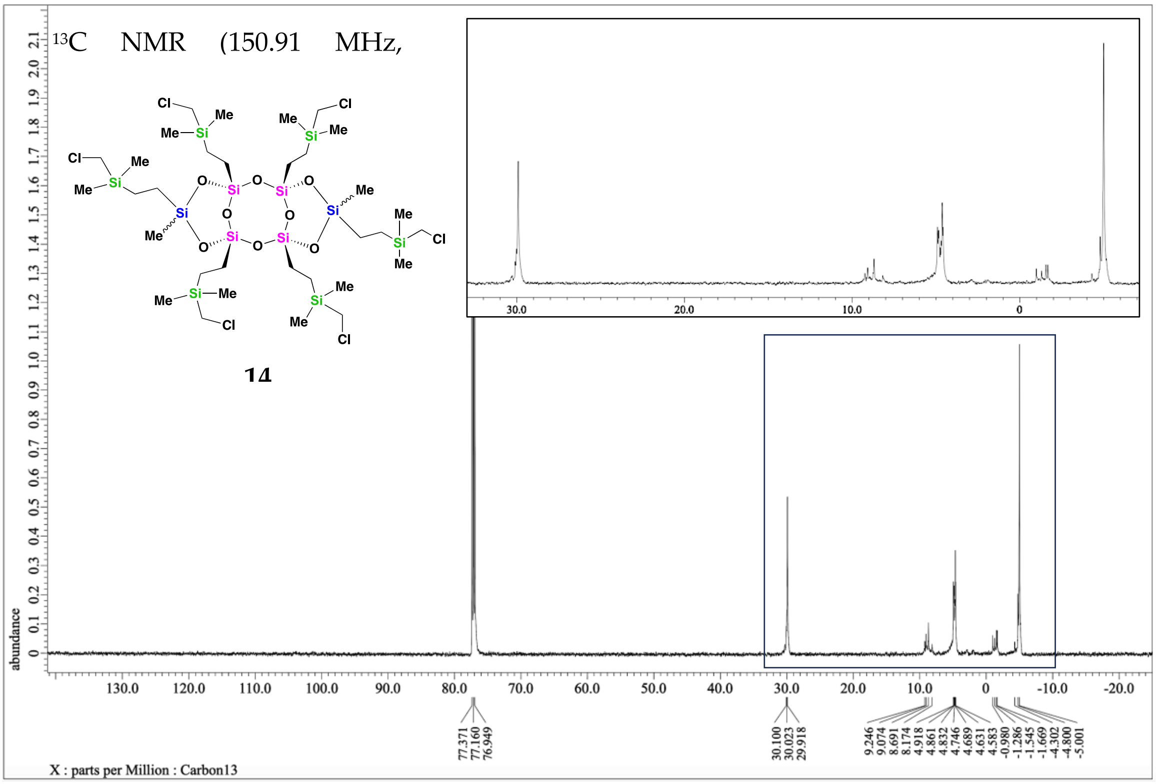

Figure S42. ^{13}C NMR spectrum for **14**.

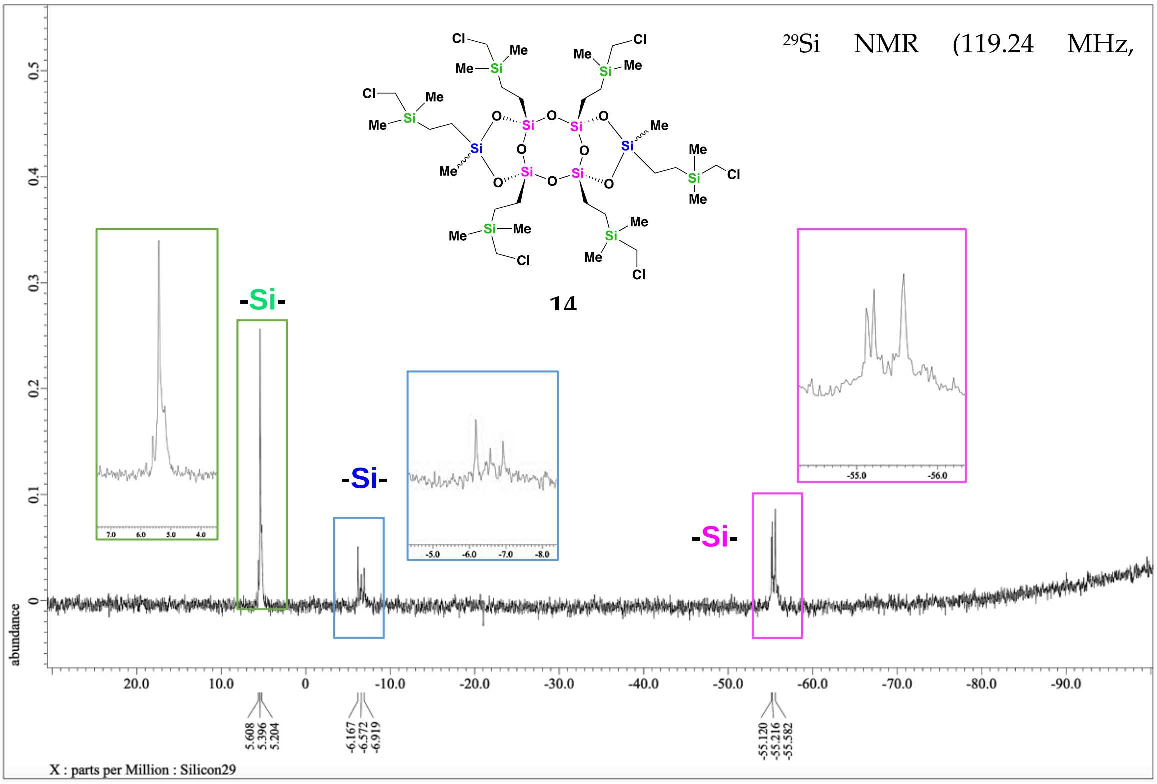

Figure S43. ^{29}Si NMR spectrum for **14**.

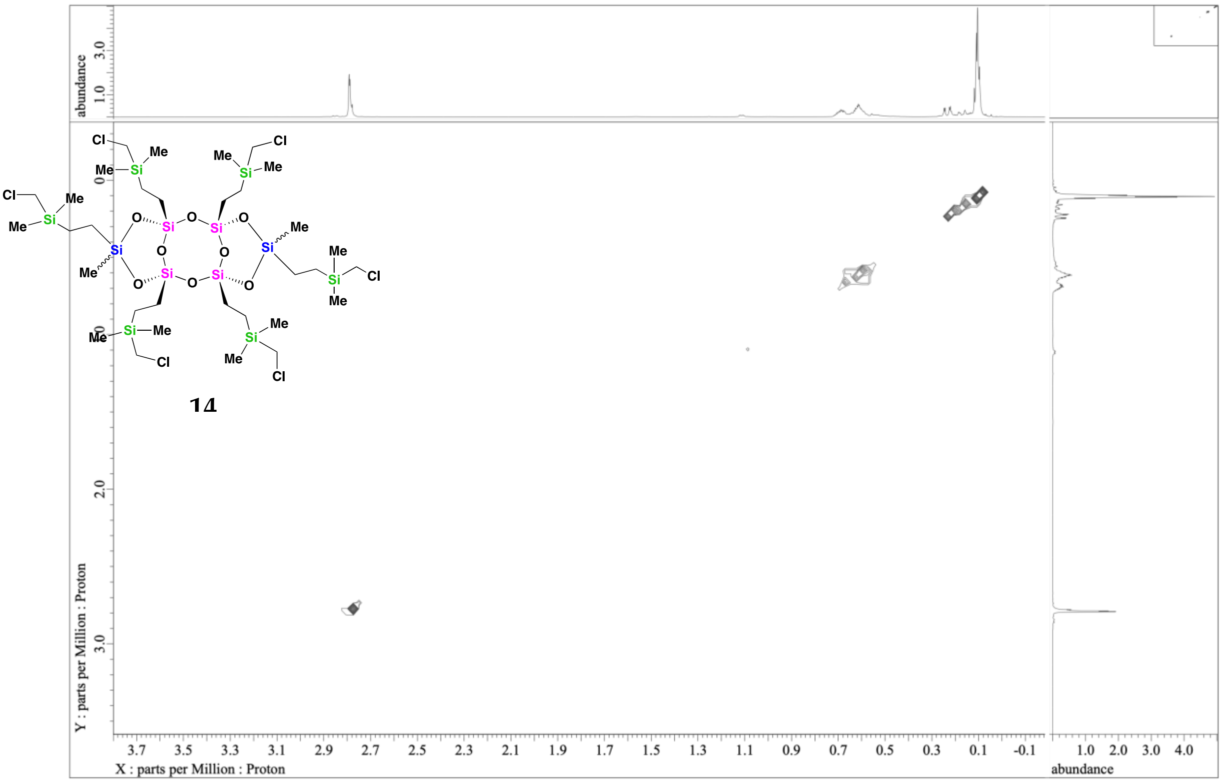

Figure S44. ^{1}H-^{1}H COSY NMR spectrum for **14**.

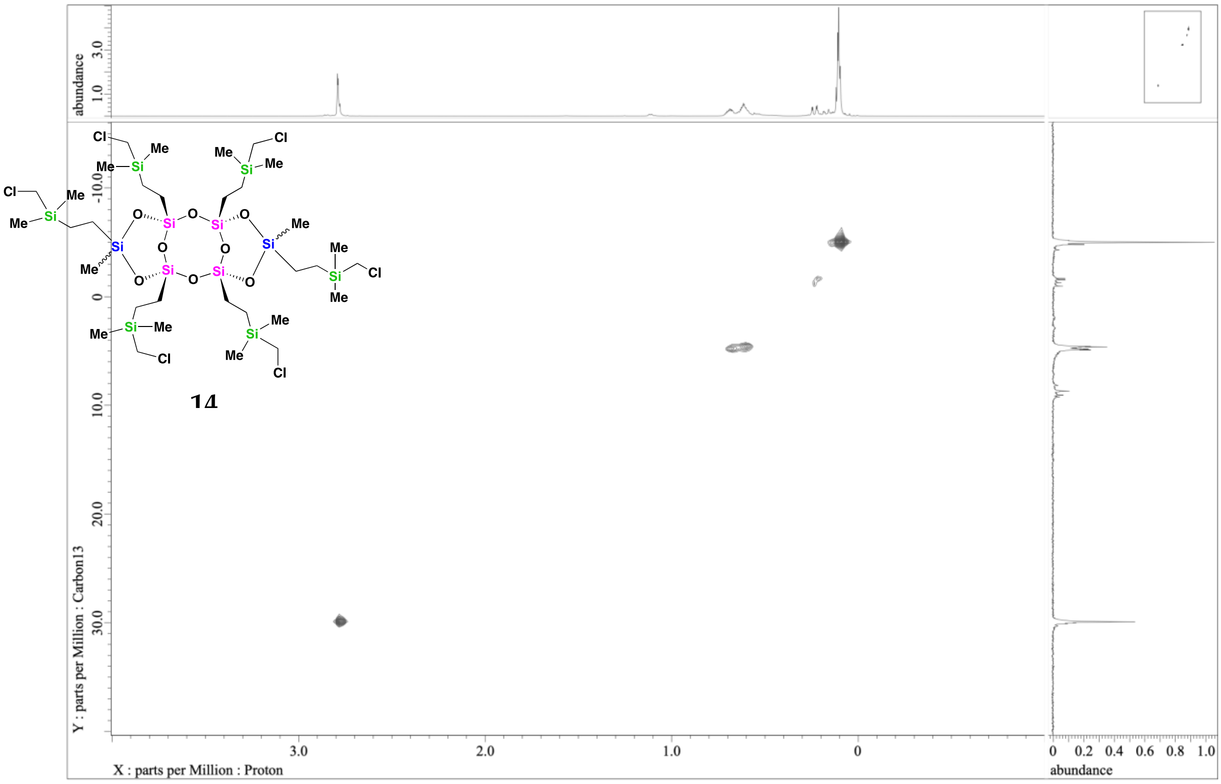

Figure S45. ^{1}H-^{13}C HSQC NMR spectrum for **14**.

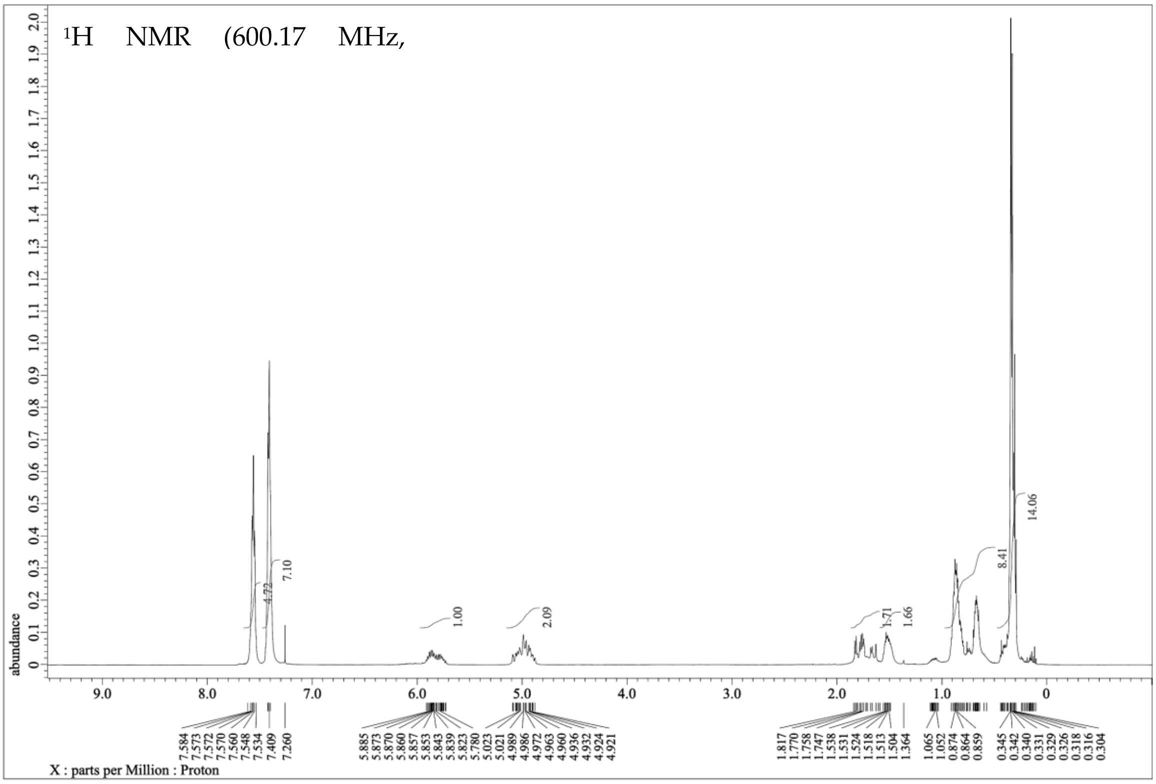

Figure S46. ^{1}H NMR spectrum for **15**.

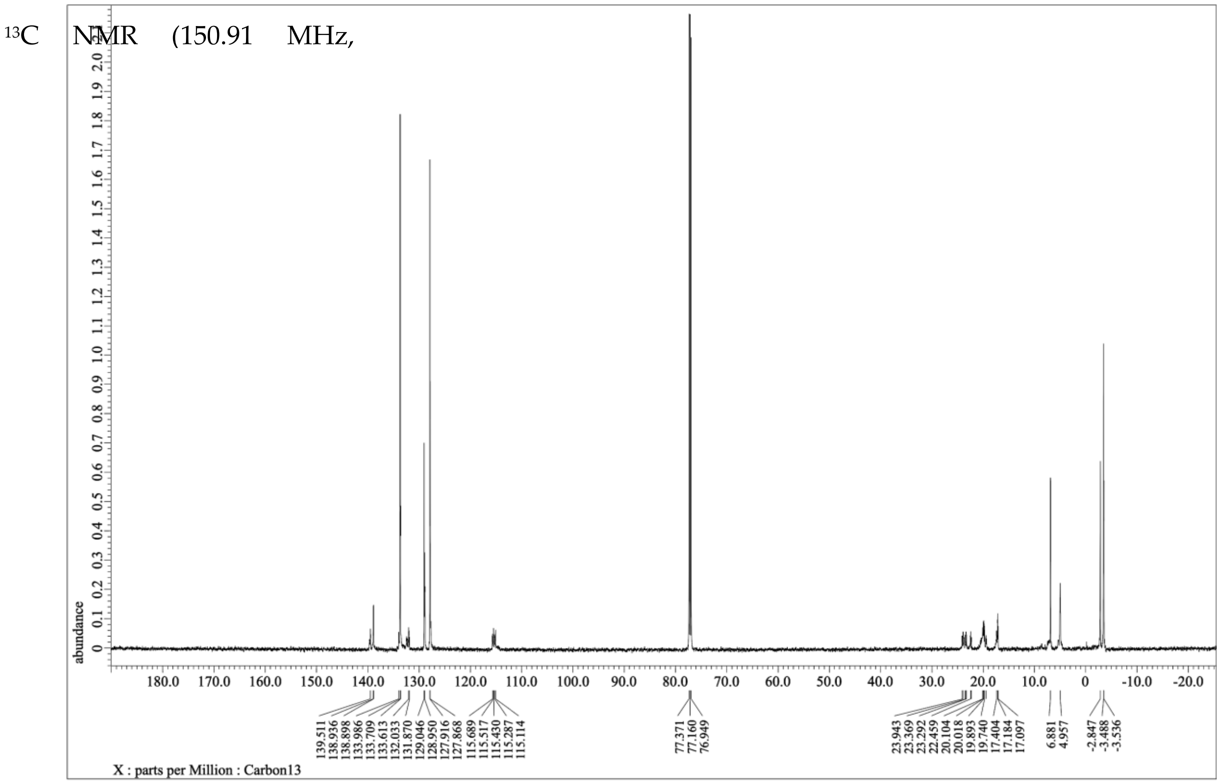

Figure S47. ^{13}C NMR spectrum for **15**.

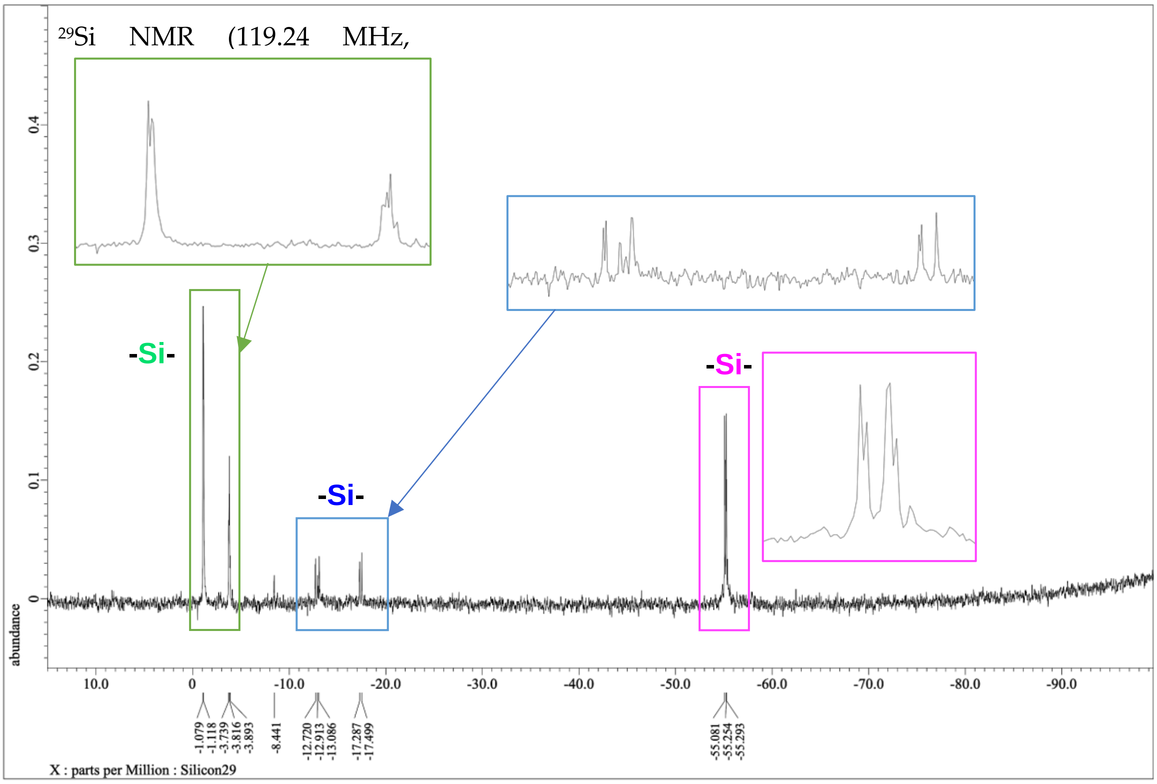

Figure S48. ^{29}Si NMR spectrum for **15**.

3. MALDI-TOF MS Spectra for synthetic compounds 6-15

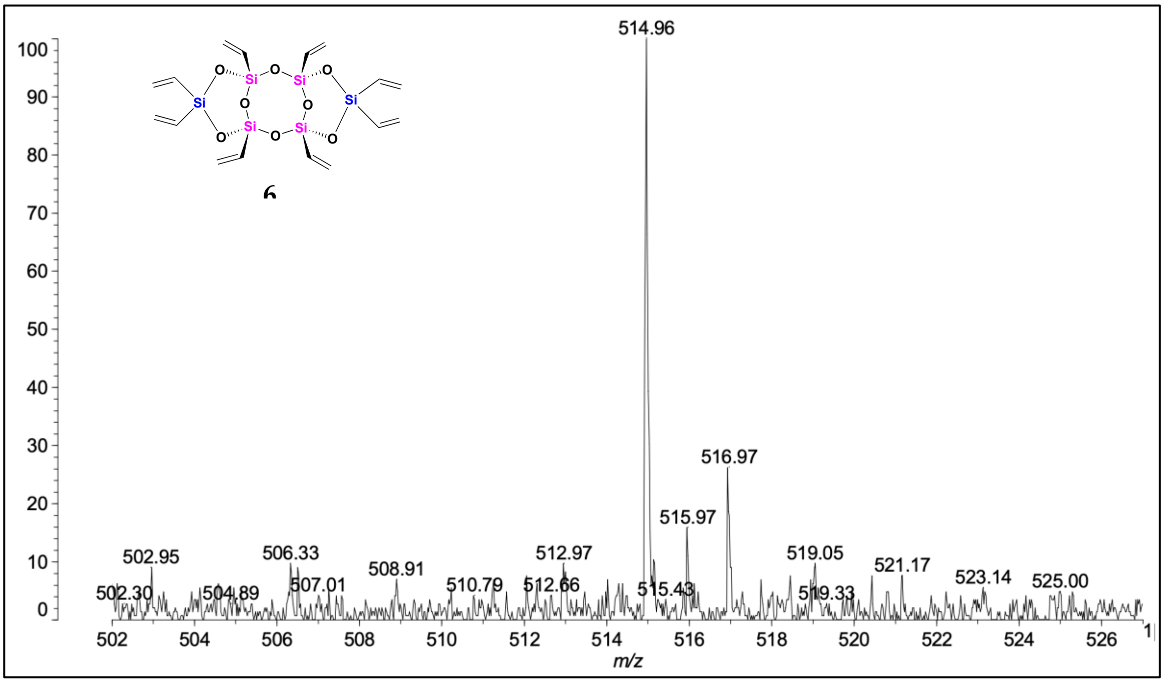

Figure S49. MALDI-TOF MS spectrum for **6**.

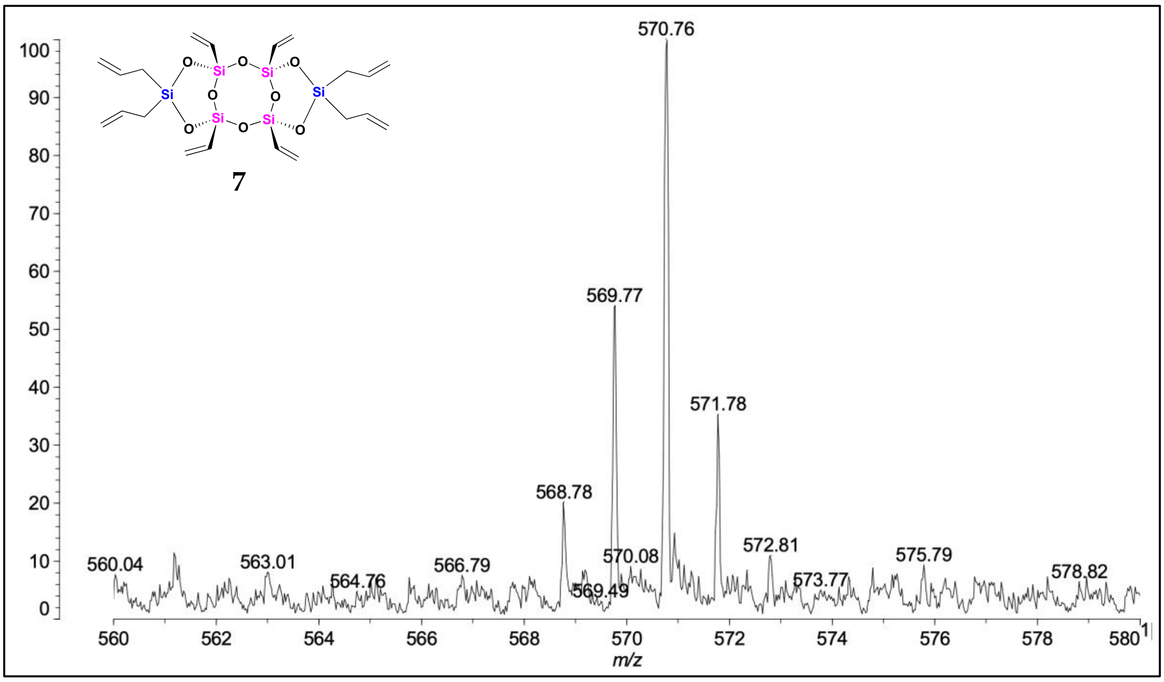

Figure S50. MALDI-TOF MS spectrum for **7**.

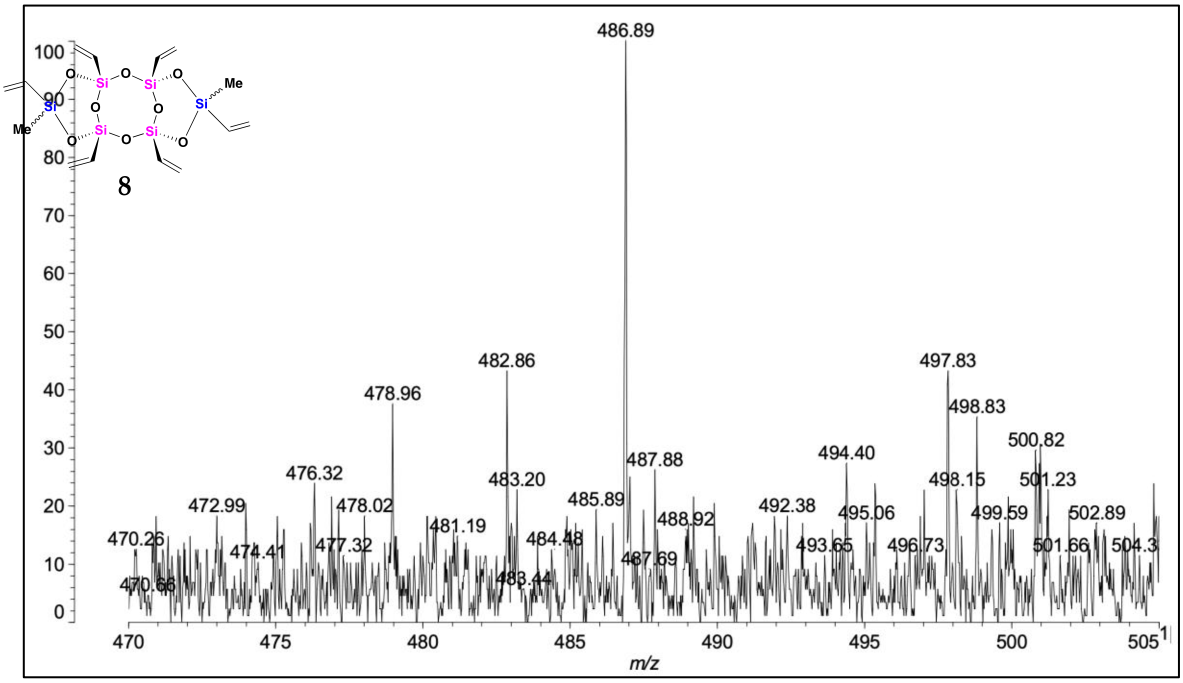

Figure S51. MALDI-TOF MS spectrum for **8**.

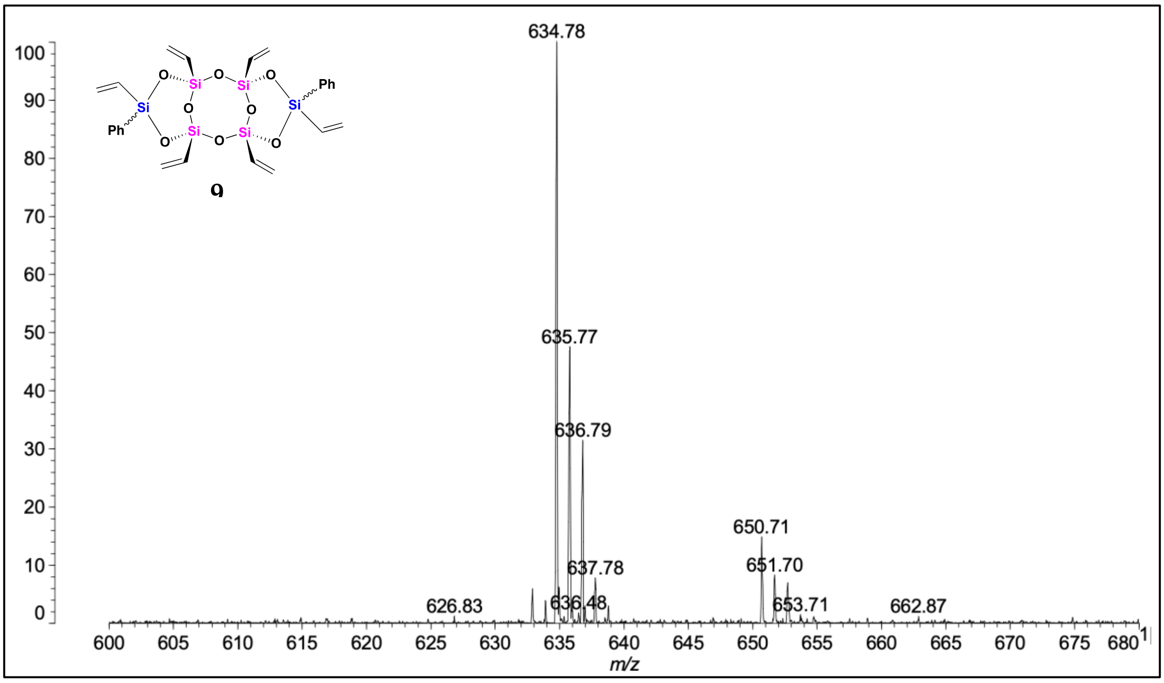

Figure S52. MALDI-TOF MS spectrum for **9**.

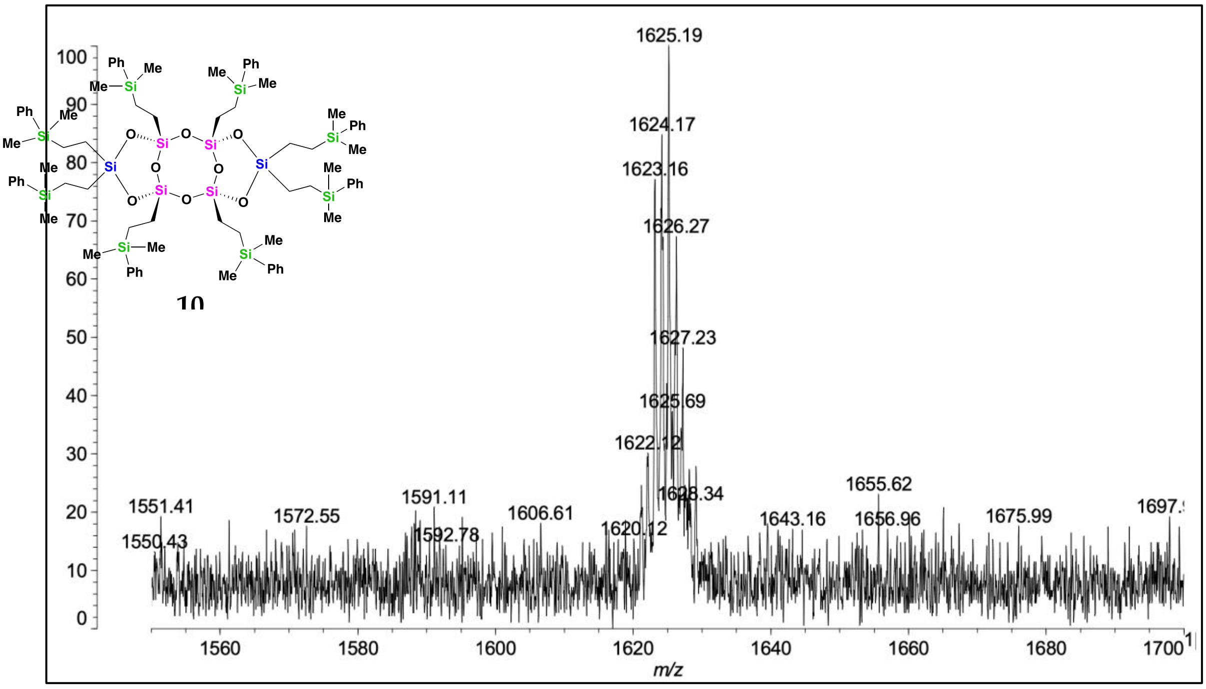

Figure S53. MALDI-TOF MS spectrum for **10**.

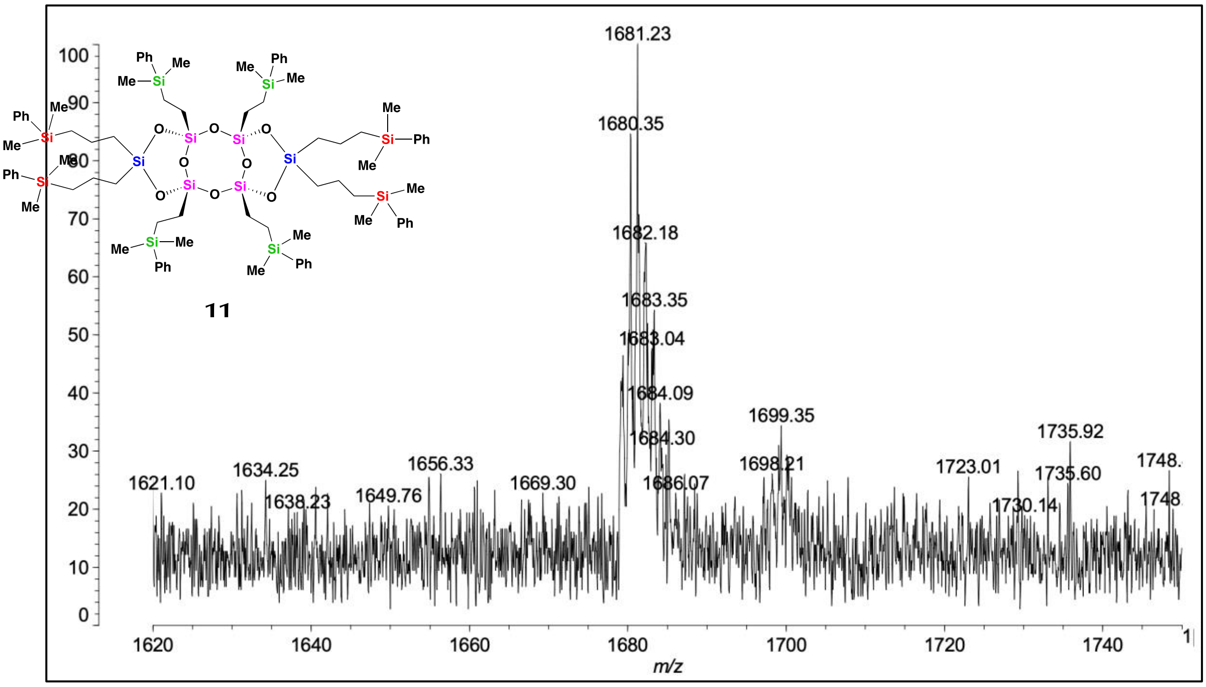

Figure S54. MALDI-TOF MS spectrum for **11**.

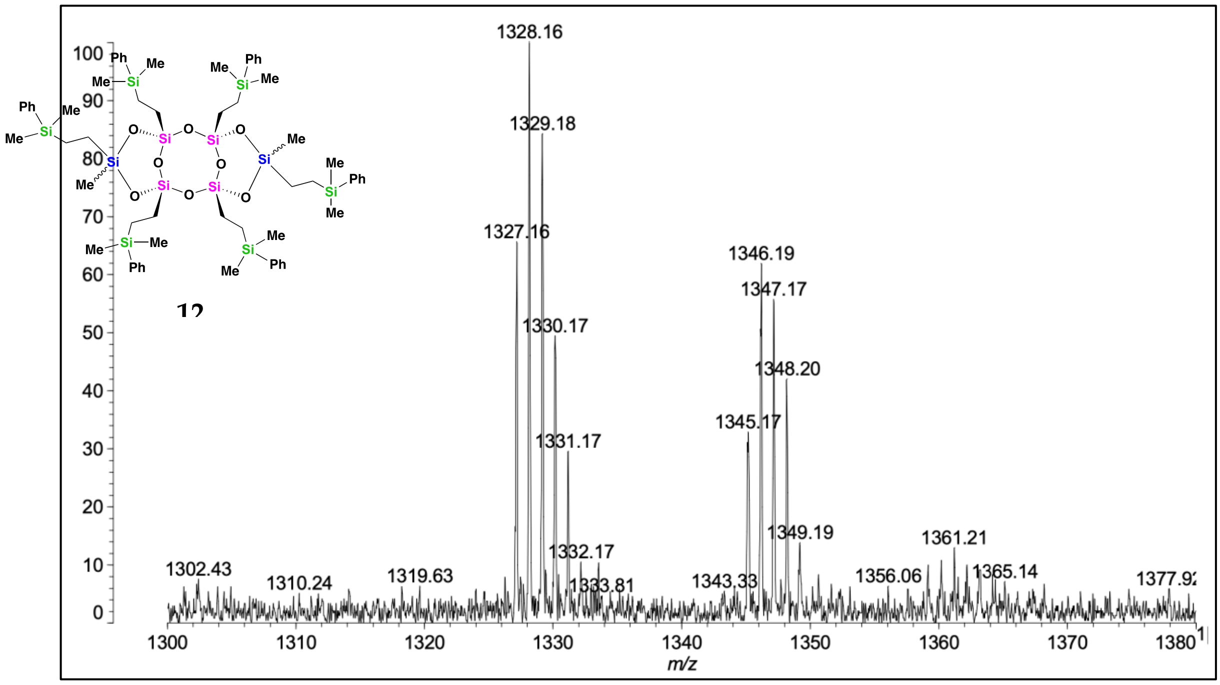

Figure S55. MALDI-TOF MS spectrum for **12**.

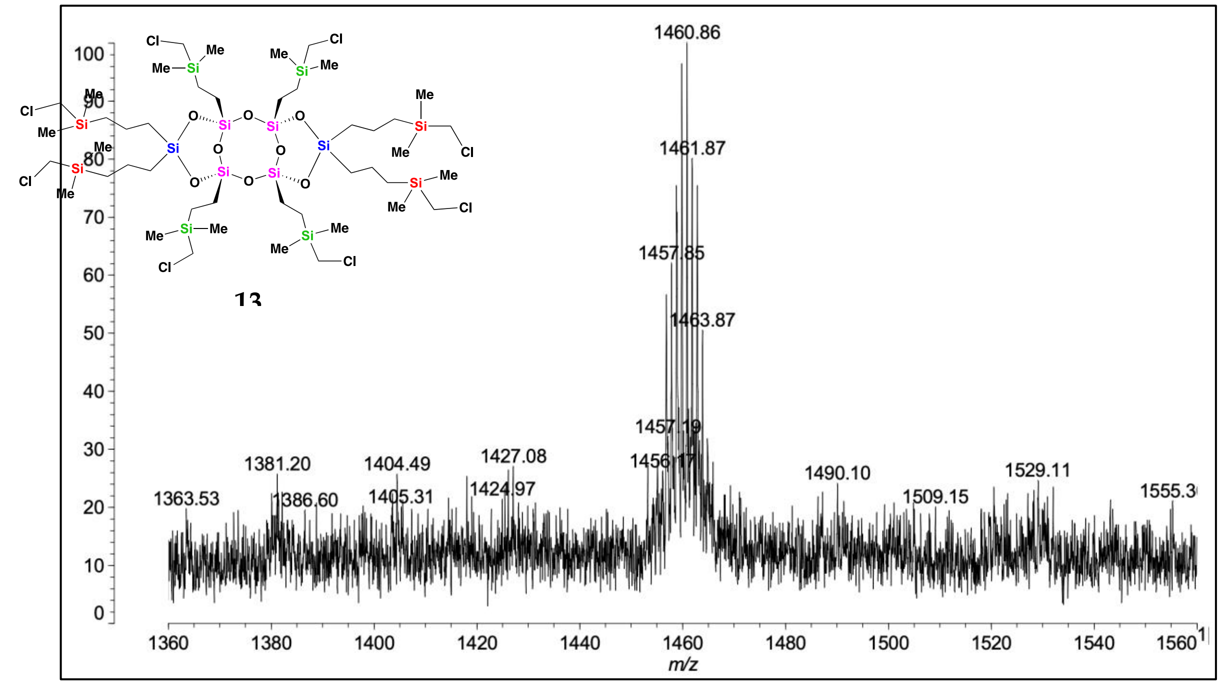

Figure S56. MALDI-TOF MS spectrum for **13**.

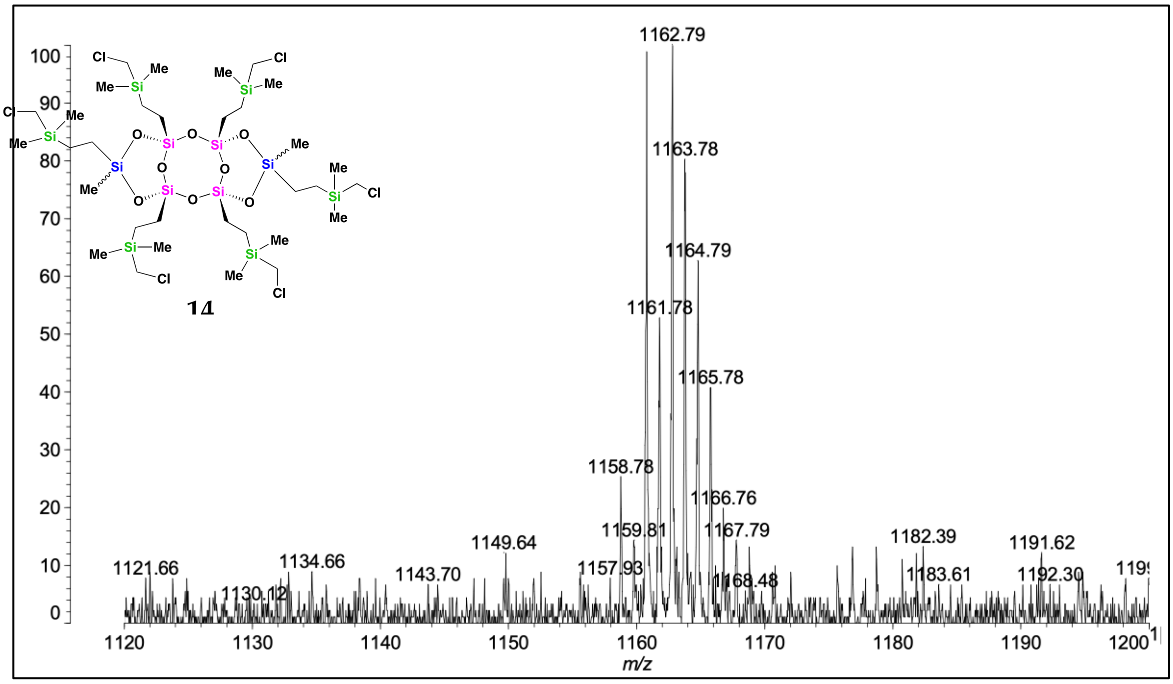

Figure S57. MALDI-TOF MS spectrum for **14**.

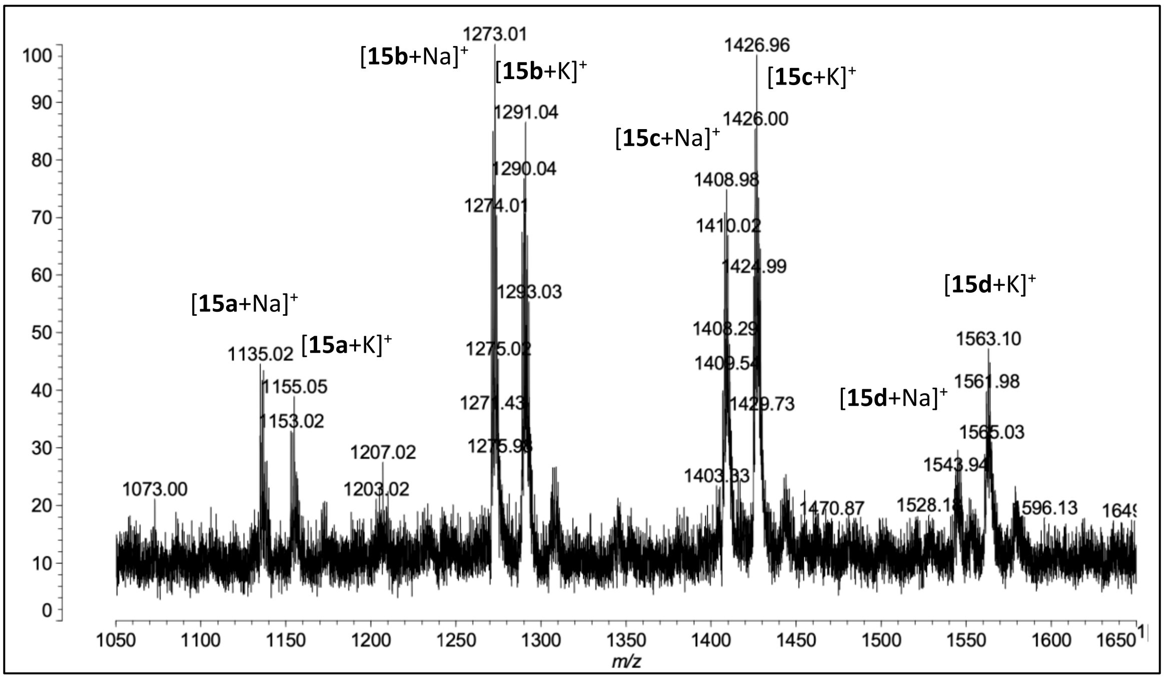

Figure S58. MALDI-TOF MS spectrum for **15**.

4. Thermogravimetry/Differential Thermal Analysis (TG/DTA) for compounds 6-14

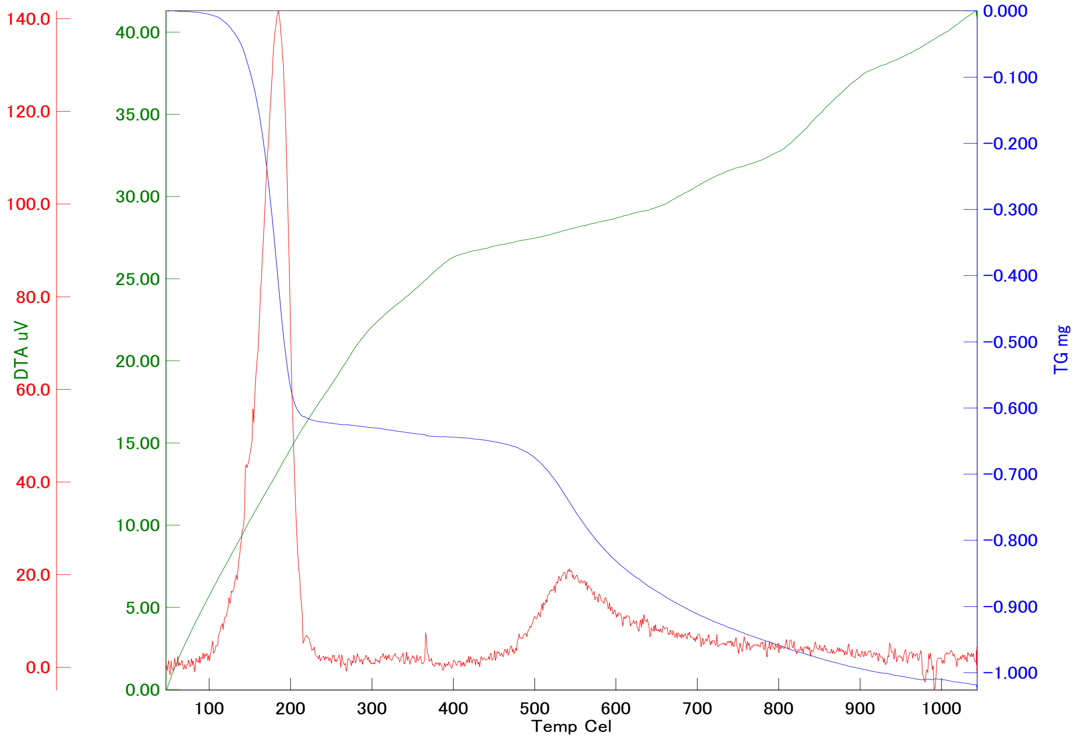

Figure S59. TG/DTA spectrum for **6** under N_2.

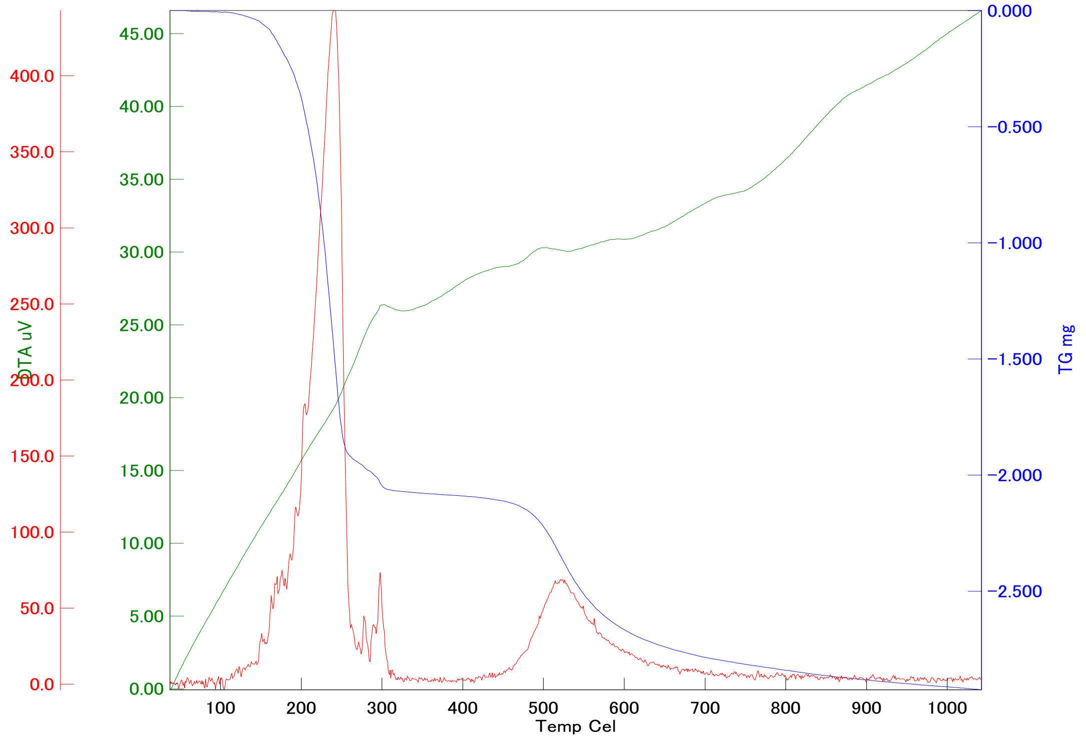

Figure S60. TG/DTA spectrum for **7** under N_2.

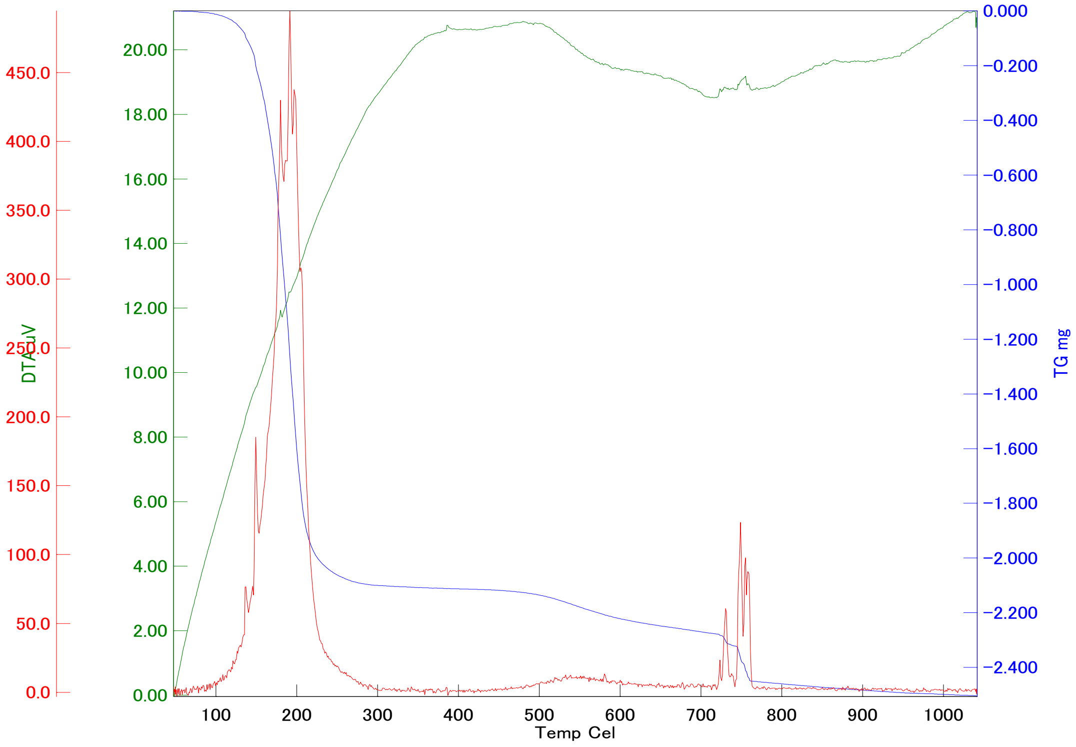

Figure S61. TG/DTA spectrum for **8** under N_2.

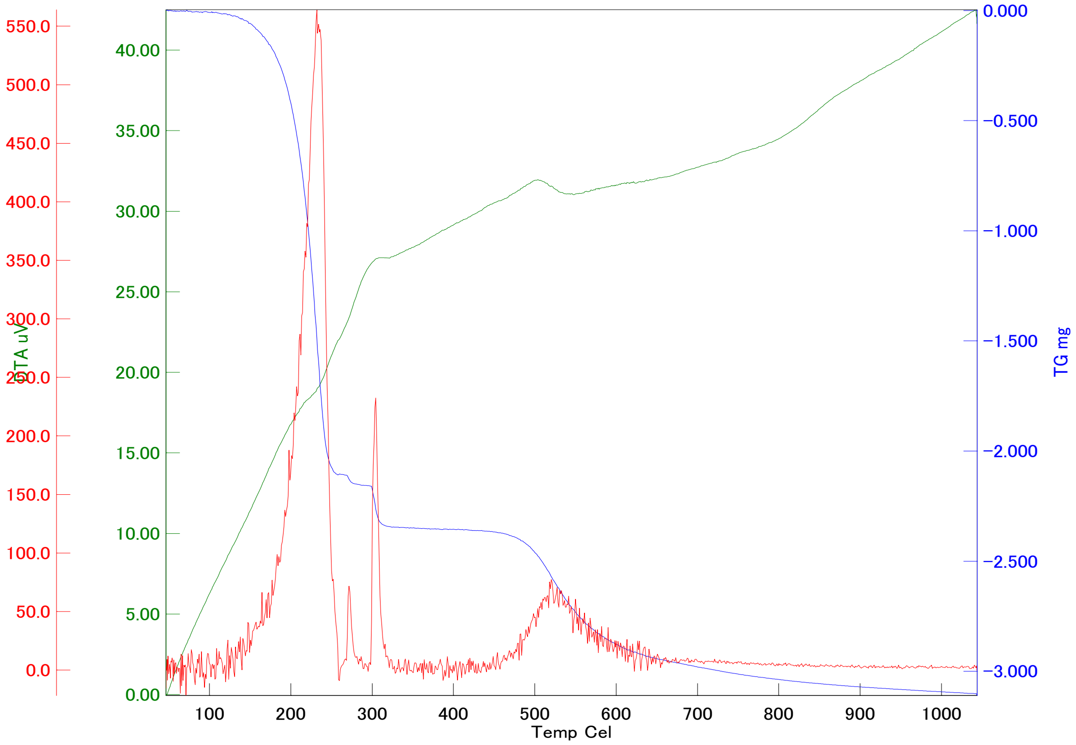

Figure S62. TG/DTA spectrum for **9** under N_2.

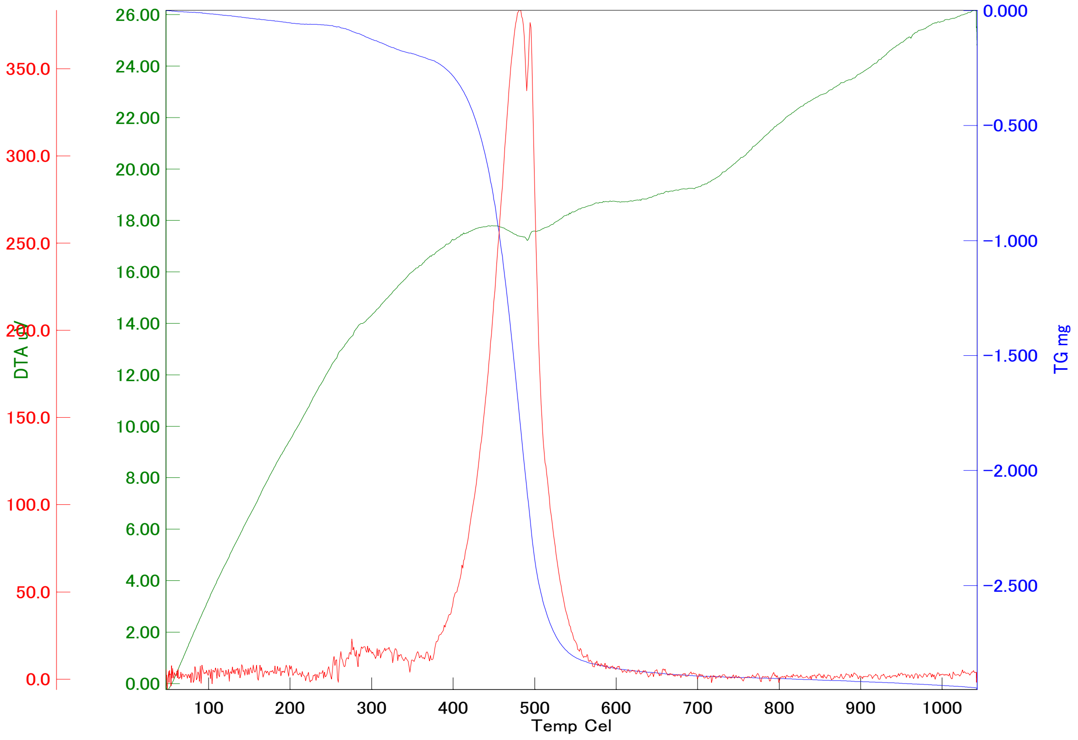

Figure S63. TG/DTA spectrum for **10** under N_2.

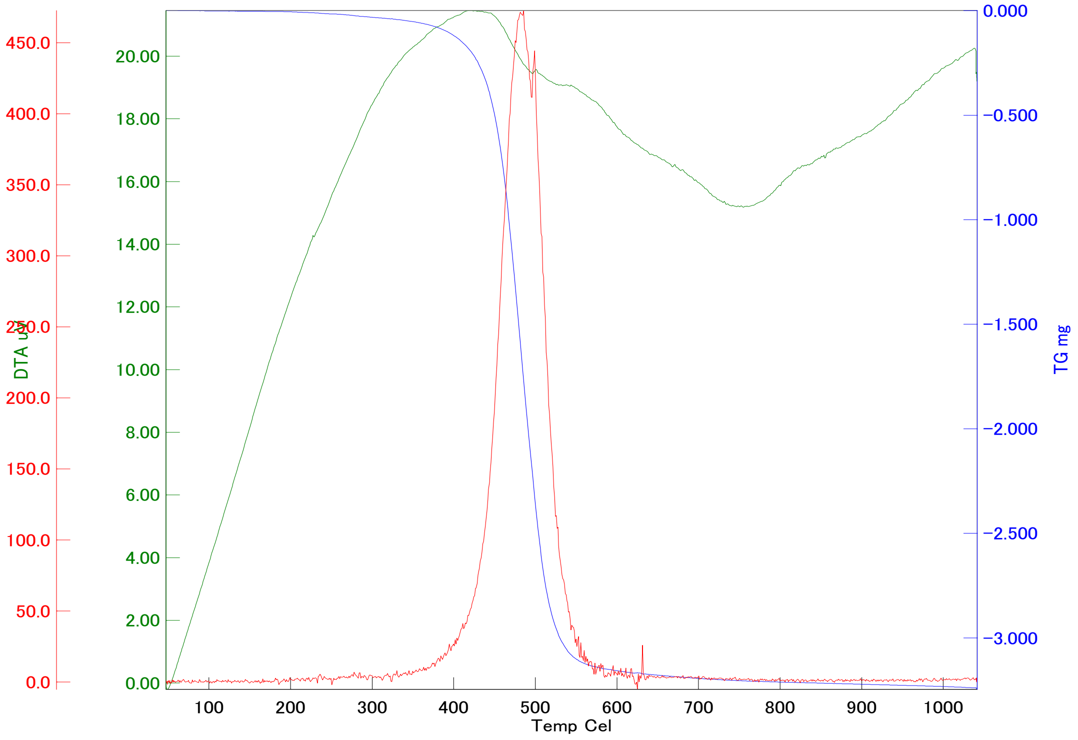

Figure S64. TG/DTA spectrum for **11** under N_2.

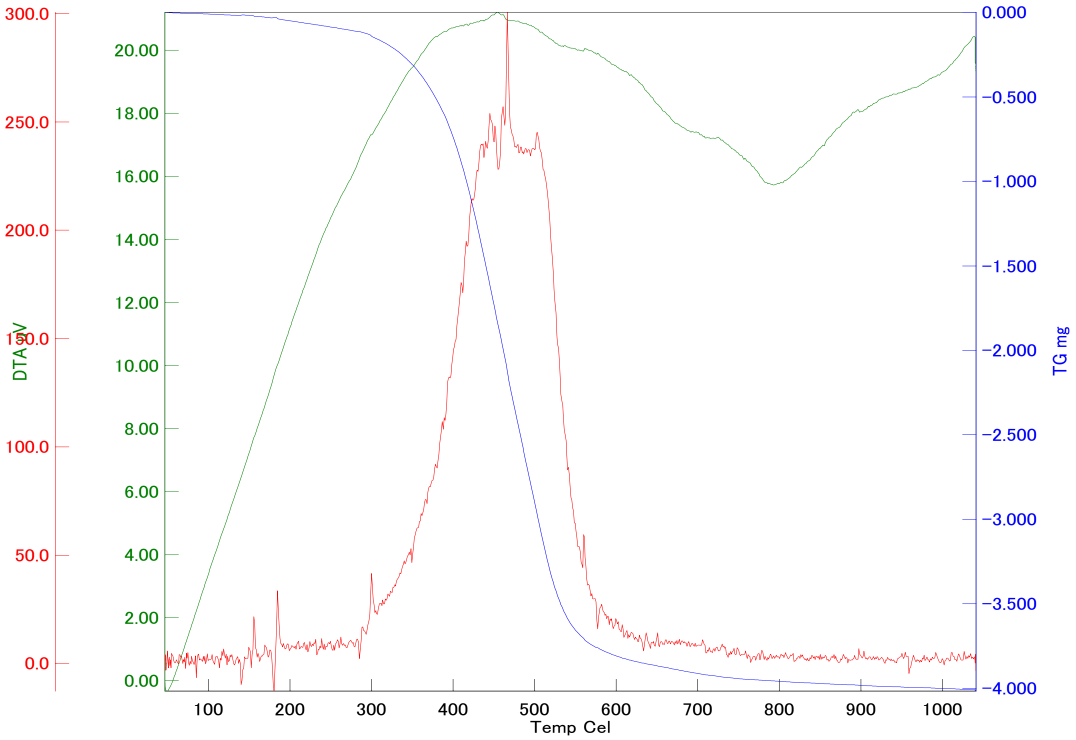

Figure S65. TG/DTA spectrum for **12** under N_2.

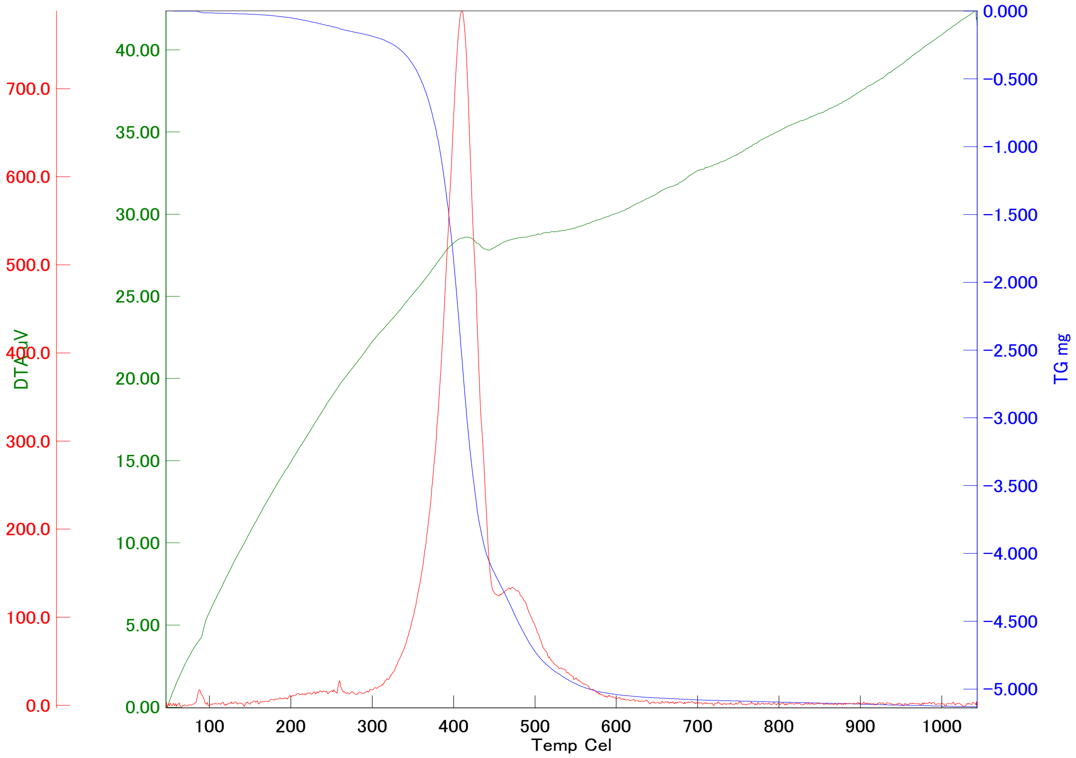

Figure S66. TG/DTA spectrum for **13** under N_2.

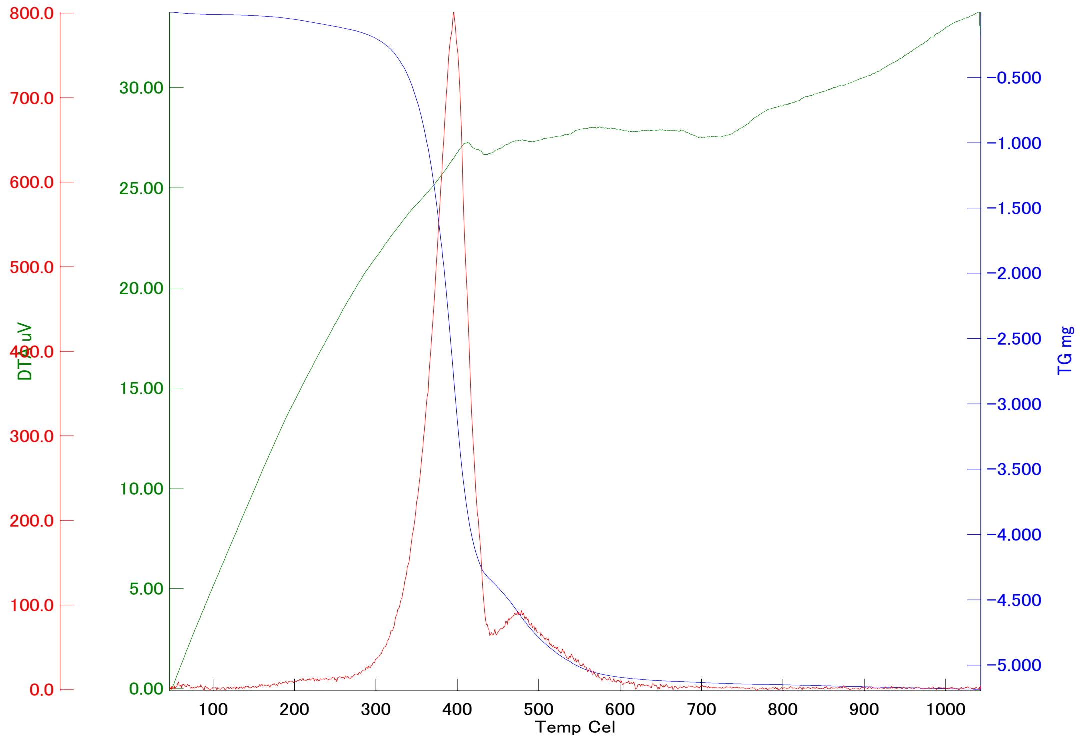

Figure S67. TG/DTA spectrum for **14** under N_2.

Table S1. Thermal properties for compounds **6-14** under N_2.

**Compounds**	**Td$_5$ (˚C)**	**Residue at 1000 ˚C (%)**
**6**	172	80
**7**	196	55
**8**	148	24
**9**	198	60
**10**	340	18
**11**	422	20
**12**	348	33
**13**	333	10
**14**	323	17
